# SPectroscopy Informed Configurational Enhanced Sampling (SPICES)

**DOI:** 10.1021/acs.jctc.6c01326

**Published:** 2026-10-23

**Authors:** Roel van de Ven, Herma Cuppen, Daria Ruth Galimberti

**Affiliations:** Institute for Molecules and Materials, 6029 Radboud University, Heyendaalseweg 135, Nijmegen 6525 AJ, The Netherlands

## Abstract

We present a new enhanced-sampling scheme for obtaining the underlying conformer starting from a spectrum: the SPectroscopy Informed Configurational Enhanced Sampling (SPICES) method. SPICES is driven by a history-dependent biased molecular dynamics, in which the biases are governed by spectral information rather than by energetic considerations. The performance of SPICES is hence rather insensitive to the target conformer’s relative energy compared to the global minimum structure. Furthermore, it does not require pretraining or prior knowledge of the system. We have critically assessed the performance of SPICES and demonstrated that it can identify the correct conformer of our test system, diphenylalanine (FF), among the 400 stable conformations in 22 spectral calculations (median value). We also show that SPICES is robust to noise in the experimental spectrum and to small discrepancies between theory and experiment.

## Graphical abstract

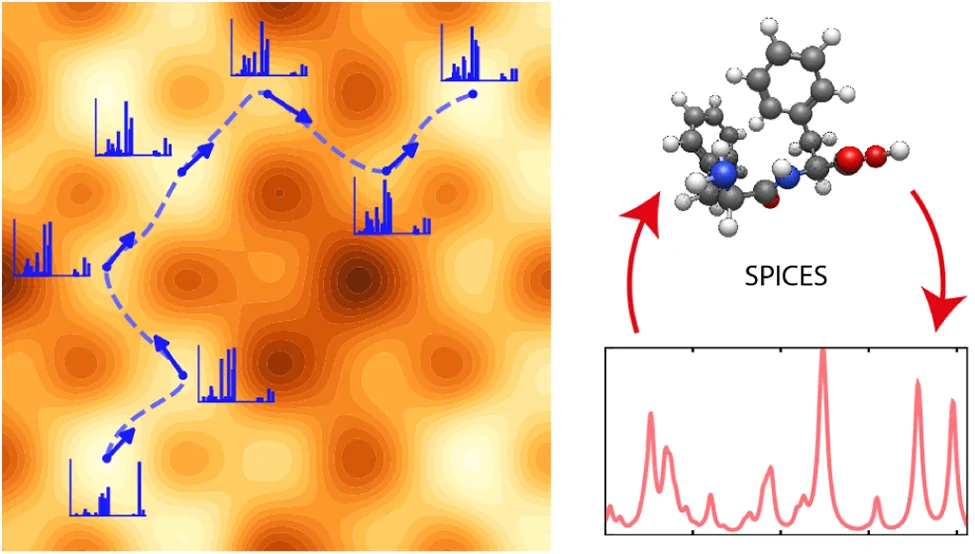

## 1 Introduction

The specific properties and functionalities of molecular materials are determined by their three-dimensional structure and the underlying interplay of intra- and intermolecular interactions, e.g., covalent bonds, hydrogen bonds, van der Waals interactions, and Coulomb interactions. To study these systems, vibrational spectroscopy techniques, such as infrared spectroscopy, are widely used as they are highly versatile and sensitive to both local and global information on the organization of the molecular system, including all intra- and intermolecular phenomena.^1–4^ However, due to the complexity of the information, translating vibrational spectra into 3D structures even for a single molecule is not always straightforward. In this regard, computational approaches are a powerful ally, helping to interpret the hidden details. The correct interpretation of an experimental spectrum requires determining the system’s structure. This chicken-and-egg problem is usually solved by mapping the Potential Energy Surface (PES), followed by calculating the spectrum for each of the optimized minima. This approach becomes increasingly problematic for larger molecules, as the conformational space grows exponentially with the number of atoms. In the past decade, the exploration of the PES has become faster thanks to efficient algorithms that can be divided into two broad classes.^5,6^ The first class contains the systematic methods, which follow particular rules to generate all possible conformers.^7–12^ The second class comprises stochastic methods that sample the conformational space. This category includes Monte Carlo simulations,^13–17^ genetic algorithms,^18–20^ and molecular dynamics–based approaches, such as metadynamics^21,22^ and replica-exchange molecular dynamics.^23–28^ For both classes, cost-effective methods to describe molecular interactions—such as classical force fields, semiempirical methods,^29–31^ machine-learning force fields (MLFFs), and neural network potentials (NNPs)^32–39^ —are commonly employed.

Nevertheless, once the minima have been efficiently identified, the corresponding spectra must be computed. Even for many medium-sized systems, it is not feasible to calculate the spectra for all identified structures—often numbering in the hundreds to thousands—since such calculations typically require a high level of theory, preferably DFT hybrid functionals with polarized basis sets.^40–47^ The computational cost imposes a practical limit on the number of spectra that can be calculated. A standard approach would use a clustering method based on collective variables to prevent spectrum calculations of structures that are too similar.^48–50^ Additionally, an energy window is typically employed to exclude high-energy structures. However, this has some drawbacks. Typically, high-level methods, i.e., large basis sets and hybrid functionals, or beyond, are needed to obtain accurate energies. If less accurate methods are used to generate the structures and evaluate their relative stability, larger energy windows (∼25 kJ/mol) have to be taken to compensate and to ensure that the correct conformer is not filtered out.^42,50^ Moreover, the energy-window approach assumes that the correct structure corresponding to the experimental measurements lies among the lowest-energy conformers. This is not necessarily true, as a molecule may be kinetically trapped in a higher-energy state. In the case of a reaction, one may be interested in identifying a (high-energy) intermediate. In the case of a crystal, the system may reside in a phase that is not the most thermodynamically stable.

Recently, to ease the computational burden of computing thousands of spectra, machine-learning strategies have been proposed for vibrational spectroscopy^34,51–60^ to reduce the computational demands of a single spectrum calculation and enable the calculation of a larger number of spectra. However, these methods are data-hungry, requiring an intensive training set (>200–1000 structures),^52^ which is not always available *a priori*. Moreover, if high-quality spectra are required, transferability across different systems is an issue.^57^ This strongly reduces the applicability or at least the advantage of these approaches.

Here, we propose an alternative approach that shifts the paradigm. This strategy was developed with three objectives in mind. i) Rather than seeking ways to reduce the cost of individual spectrum calculations, we focused on minimizing the number of required calculations. Ultimately, only the correct conformer is of interest, whereas performing expensive calculations for all other candidates provides a limited added value and represents a significant waste of computational resources. ii) The second objective is to avoid using energies as a selection criterion for candidate structures. iii) The third objective is to develop an approach that does not require any prior knowledge (or a training set) for a given system.

In recent years, enhanced-sampling techniques, such as path metadynamics, have been shown to be powerful tools for chemical reactions and phase transitions.^61–65^ We draw inspiration from this class of techniques to develop a new type of enhanced-sampling molecular dynamics, governed by spectral-matching criteria rather than energetic considerations: spectroscopy-informed configurational enhanced sampling (SPICES).

Section 2 illustrates the method. Section 3 reports the computational details for the benchmark system. Section 4 discusses the performance of the different strategies and configurations.

## 2 Method and Approach

### 2.1 Definition of the Problem

The vibrational spectrum of a system is a continuous function, which can be discretized and represented as a vector ***I*** (see Section 2.4). For any system, one can define a set of coordinates {*q*_*i*_} (e.g., Cartesian, internal coordinates, or other descriptors) whose values, {*Q*_*i*_}, uniquely identify the system’s structure. We denote the vector collecting these values as ***Q***. Specifically, ***Q*** (*t*) represents the coordinates at step *t* of the simulation, while ***Q*** ^*X*^ denotes the coordinates corresponding to a specific state *X*. Finally, we define I as a correspondence between the coordinate space and the spectral space, where $I\left(Q^{X}\right)=I^{X}$ represents the spectrum associated with a specific configuration ***Q*** ^*X*^.

The goal here is to devise a method that identifies the structure(s) ***Q*** * that correspond to the target spectrum ***I*** *, i.e., finds ***Q*** * for which $I\left(Q^{*}\right)=I^{*}$ , given a reference state 0 for the system with known structure ***Q*** ^0^ (e.g., a conformation of a molecule, a cluster shape, a phase of a crystal) and spectrum ***I*** ^0^. As stated in the introduction, we further require that the method finds the solution of this inverse problem without complete sampling of the potential energy surface (PES) and without any prior knowledge of the PES.

When developing such a method, it is essential to keep in mind that although each structure ***Q*** corresponds to a unique spectrum—i.e., the mapping I(Q) is well defined when {*q*_*i*_} is a complete set for the system’s structure—the relation is not bijective, i.e., the inverse relation is not one-to-one. In general, several distinct structures ***Q*** may produce the same spectrum. As a consequence, strategies based on a simple mapping of a Q(I) functional relation are not the best approach because, in general, such a field does not exist or is ill-defined.

Because addressing this inverse problem requires a framework capable of producing multiple solutions with an associated statistical distribution, we take inspiration from statistical-thermodynamics methods commonly employed in the analysis of chemical reactions and phase transitions. Accordingly, we reformulate the initial problem as the identification of a path S , for which

1 $$I^{*}=I^{0}+\int_{S}\nabla IdQ$$

S does not need to be unique, and therefore, multiple solutions can be handled and sampled. In the case of a discretized spectra $\left(I\in R^{n}\right)$ , ∇ ***I*** is naturally identified with the Jacobian matrix of ***I*** with respect to $Q\left(Q\in R^{m}\right)$ . Its elements can be written explicitly as

2 $${\left(J_{I}\right)}_{i,j}=\frac{\partial I_{i}}{\partial Q_{j}},,i=1,...,n,j=1,...,m$$

and

3 $$dI=J_{I}\left(Q\right)dQ$$

The functional form of ∇ ***I*** is unknown, and therefore, an analytical solution is impossible. However, the problem can be solved iteratively using numerical methods, as described below. The system can be seen as a walker that explores the ***I*** (***Q***) space. At each iteration, *n*, the walker makes a move, i.e., it changes its ***Q*** of a certain discrete amount, Δ ***Q***, and, as a consequence, its spectrum changes. The walker progresses through configuration space—thus generating a path S —until it reaches a point ***Q*** for which the computed spectrum ***I*** (***Q***) coincides with the target spectrum ***I*** *. Notice that for each walker’s move, a new spectrum must be computed. Because computing spectra is expensive, ideally, the system evolution should be designed to favor short paths. To this end, we develop a novel importance-sampling scheme described below. To limit the computational cost, we treat a walker’s move as a jump between minima of the PES. In this case, the spectra need to be evaluated only for these minima positions. An additional advantage of this strategy is that the set of coordinates {*q*_*i*_} now must serve merely as a descriptor to uniquely define only the minimum-energy structures instead of all possible structures, which results in a considerable reduction in the dimensionality of the space. Finally, a further advantage is that to be able to use the harmonic approximation to compute the spectra, one needs to be at a minimum.

Initially, there is no prior information regarding the PES or its minima; thus, the walker must be able to discover these points during the simulation. To this end, we devised a biased molecular dynamics approach based on a history-dependent configurational displacement field, ***s***_*n*_(***I*** *, ***I*** ^*n*^, ***Q*** ^*n*^|{***I*** ^ref^, ***Q*** ^ref^}), hereafter denoted in short as the “history-dependent displacement field”. The ***s***_*n*_ field directs the walker toward the target spectrum ***I*** * by taking the current state (***Q*** ^*n*^, ***I*** ^*n*^) as input and depends parametrically on the set of previously explored minima {***I*** ^ref^, ***Q*** ^ref^}. This spectrum-directed enhanced sampling method will be called SPectroscopy Informed Configurational Enhanced Sampling (SPICES) from here on and is schematically depicted in Figure 1. We will walk through the individual steps in detail below.

**Figure 1:**
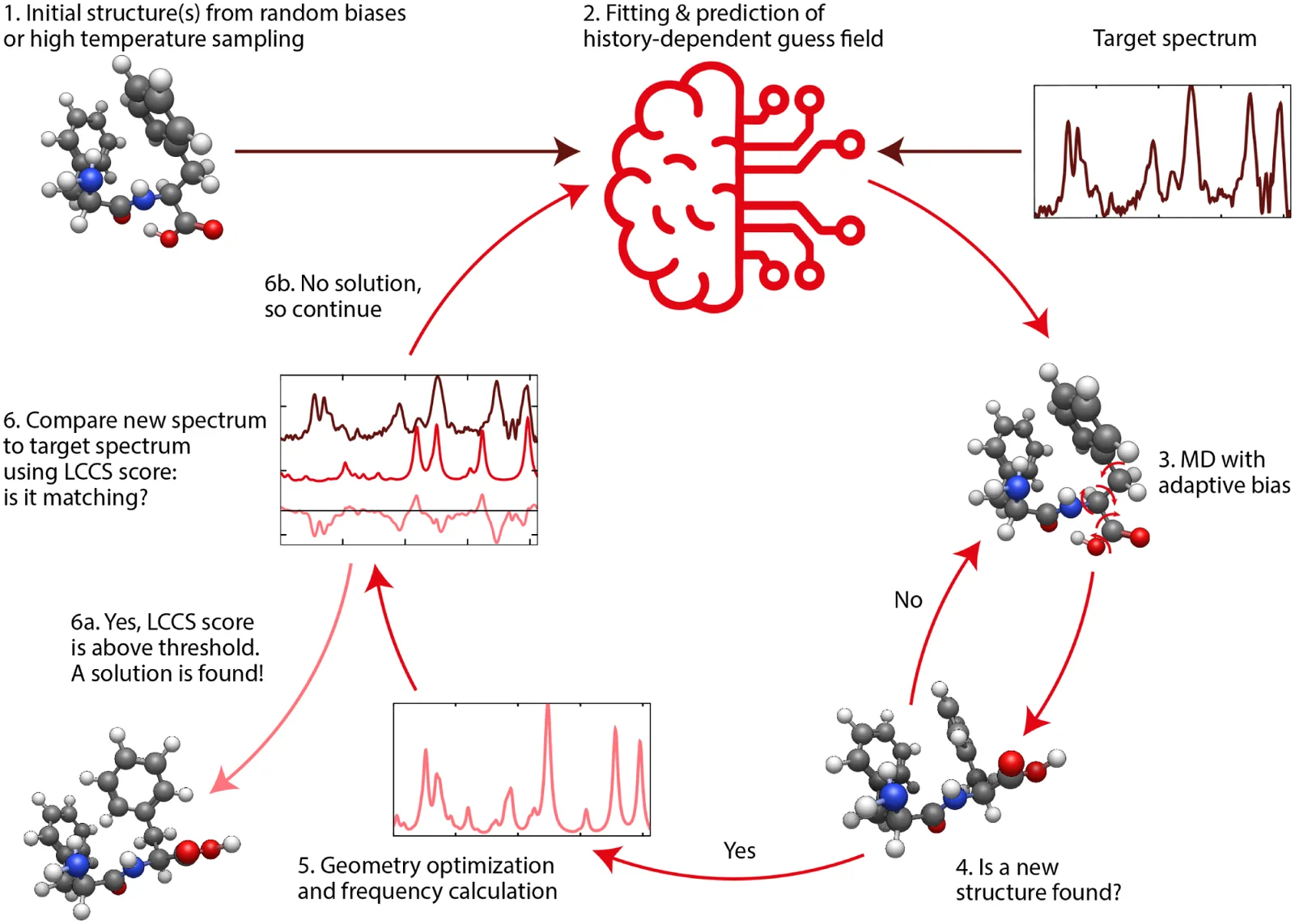
Schematic overview of the SPectroscopy Informed Configurational Enhanced Sampling (SPICES) workflow. The machine-learning logo was taken from https://www.flaticon.com/free-icons/machine-learning.

Step 1: The procedure starts by providing a random initial structure and selecting the set of coordinates {*q*_i_} whose values {*Q*_i_} are used in SPICES. We create a few reference structures by random bias or high-temperature sampling (see Section 2.8). Step 2: Using the spectra computed for these structures, we build the history-dependent displacement field, ***s***_*n*_ (Section 2.7). Step 3: ***s***_*n*_ is used as a driver in a molecular dynamics simulation with an adaptive bias to generate a new, promising structure based on the target spectrum (Section 2.2). Steps 4 and 5: When a possible structure is identified, its geometry is optimized and its spectrum is computed (Section 2.4). Step 6: The spectrum is then compared with the target to check whether a new structure is indeed found (Section 2.3). If not, then the information is added to the reference set, and the procedure starts again from Step 2.

### 2.2 Steering through Conformational Space

At the start of each MD with adaptive bias (step 3), ***s***_*n*_ provides a guessed linear path (***Q*** * – ***Q*** ^*n*^ ≈ ***s***_*n*_) in ***Q*** space to move from ***I*** ^*n*^ to the target spectrum ***I*** *. ***s***_*n*_ is then used by a biased molecular dynamics (MD) simulation as a compass, indicating the direction in which the system should evolve to locate the next candidate. Compared with directly applying the geometrical displacements suggested by ***s***_*n*_, this MD-based strategy offers two key advantages. On the one hand, the MD-based strategy permits the use of any complete set of {*q*_*i*_}—including redundant ones—thereby simplifying the definition of the coordinates, especially for large molecular systems. On the other hand, it compensates for the approximate nature of ***s***_*n*_ due to the limited number of reference structures and the ill-defined nature of the problem. To understand this ill-defined nature, consider Q as the inverse correspondence between the spectral space and the coordinate space such that $Q\left(I^{X}\right)=Q^{X}$ . As different structural configurations can yield identical spectra, $Q\left(I^{X}\right)$ might not be unique. The MD will lead the system to a stable structure that, following one possible mapping, correspond to the target spectra. Different history-dependent displacement fields, starting from different initial structures, can result in a different mapping of $Q\left(I^{X}\right)$ , giving different paths and multiple final solutions.

Based on ***s*** *_n_,* the parameters of a biasing potential, *V* ^*n*^(*t*, ***Q***), are set as follows:

4 $$V^{n}\left(t,Q\right)=\underset{i}{\sum }V_{i}^{n}\left(t,Q_{i}\right)$$

with *V*_*i*_ being the component of the biasing potential acting on the *q*_*i*_ coordinate. Each *V*_*i*_ has the shape of an asymmetric ratchet-and-pawl-like repulsive potentials,^66–69^

5 $$V_{i}^{n}\left(t,Q_{i}\right)=\{\begin{array}{ll} \frac{1}{2}k_{i}^{n}{\left(\rho_{i}(t,Q_{i})-\rho_{i}^{\min}(t,Q_{i})\right)}^{2} & \rho_{i}\left(t,Q_{i}\right)>\rho_{i}^{\min}\left(t,Q_{i}\right)\\ 0 & \rho_{i}\left(t,Q_{i}\right)\leq \rho_{i}^{\min}\left(t,Q_{i}\right) \end{array}$$

where

6 $$\rho_{i}\left(t,Q_{i}\right)={\left(Q_{i}(t)-{\mathrm{Q̅}}_{i}\right)}^{2}$$

and

7 $$\rho_{i}^{\min}\left(t,Q_{i}\right)=\underset{0<\tau <t}{\min}\rho \left(\tau ,Q_{i}\right)$$

*Q̅*_*i*_ is the target value imposed for the coordinate *q*_*i*_. The set of biases is applied to the walker during a molecular dynamics simulation to guide it along the defined test path and find a new conformation for the system. The strengths $k_{i}^{n}$ of the bias potential must be carefully chosen. One wants to find the next minimum as fast as possible. However, the walker needs to move along the path slowly enough that the coordinates of the system that are not in the selected {*q*_*i*_} set have time to relax. Because no prior knowledge of the potential energy surface (PES) is available, it is hardly possible to set optimal parameters for the bias potential. We therefore define $V_{i}^{n}\left(t,Q_{i}\right)$ to be adaptive.

The molecular dynamics simulation is divided into macrosteps, Γ_*j*_, consisting of *N* MD time steps. At the start of each macrostep, the target values *Q̅*_*i*_ and the potential strengths *k*_*i*_ are redefined. Macrosteps are continued until a new unexplored minimum is found (steps 3 and 4). During each Γ_*j*_ macrostep, the walker has as a goal to move a δ ***Q̅*** ^*n*^ fraction of the total path ***s*** *_n_,*

8 $$\delta {\mathrm{Q̅}}^{n}=C^{r}s_{n}$$

where ***C*** ^*r*^ are user-defined parameters dependent on the type of coordinates (e.g., angles, torsions, covalent bonds, coordination numbers) on which the changes are imposed. At the start of the first macrostep (Γ_1_), the parameters of {*V*_*i*_} (the potential strengths, {*k*_*i*_}, and the target value*s*, {*Q̅*_*i*_}) are set to

9 $$k^{n}\left(\Gamma_{1}\right)=C^{k}s_{n}$$

10 $${\mathrm{Q̅}}^{n}\left(\Gamma_{1}\right)=Q\left(\Gamma_{1}\right)+\delta {\mathrm{Q̅}}^{n}$$

where ***C***_*k*_ are again user-defined parameters dependent on the type of coordinates. ***Q*** (Γ_1_) is the value of ***Q*** at the time at which the Γ_1_ macrostep starts.

Each *q*_*i*_ coordinate is then treated separately, and the new parameters are chosen on the basis of what happened during the previous macrostep. If during the macrostep Γ_*j*–1_ the target value for *Q*_*i*_ (i.e., ${\mathrm{Q̅}}_{i}^{n}\left(\Gamma_{j-1}\right)$ ) has not been reached, the barrier to reach this target is, most likely, too high for the current potential strength $k_{i}^{n}\left(\Gamma_{j-1}\right)$ . Therefore, the potential strength is increased by a factor $C_{i}^{dk}$ for the next macrostep. If, instead, the target ${\mathrm{Q̅}}_{i}^{n}\left(\Gamma_{j-1}\right)$ is passed during the macrostep, the target is updated for the next macrostep by adding another step $\delta {\mathrm{Q̅}}_{i}^{n}$ to it, and the potential strength *k*_*i*_ is reduced by the factor $C_{i}^{dk}$ to slow the walker and prevent the bias from becoming too strong. Finally, in the case that the system moved so fast on the PES that also the value ${\mathrm{Q̅}}_{i}^{n}\left(\Gamma_{j-1}\right)+\delta {\mathrm{Q̅}}_{i}^{n}$ has been explored during the macrostep, the target ${\mathrm{Q̅}}_{i}^{n}\left(\Gamma_{j}\right)$ is set at the most extreme value explored, $Q_{i}^{ex}\left(\Gamma_{j-1}\right)$ , plus an additional step $\delta {\mathrm{Q̅}}_{i}^{n}$ , to prevent the target from being set in the “reverse” direction. At the same time, the potential strength is set back to its initial value, i.e., $k_{i}^{n}\left(\Gamma_{1}\right)$ . In particular, the following rules are used to update ${\mathrm{Q̅}}_{i}^{n}\left(\Gamma_{j}\right)$ :

11 $${\mathrm{Q̅}}_{i}^{n}\left(\Gamma_{j}\right)=\{\begin{array}{ll} Q_{i}^{\mathrm{ex}}\left(\Gamma_{j-1}\right)+\delta {\mathrm{Q̅}}_{i}^{n} & \delta \Lambda_{i}^{n}\left(\Gamma_{j-1}\right)>\delta {\mathrm{Q̅}}_{i}^{n}\\ {\mathrm{Q̅}}_{i}^{n}\left(\Gamma_{j-1}\right)+\delta {\mathrm{Q̅}}_{i}^{n} & 0<\delta \Lambda_{i}^{n}\left(\Gamma_{j-1}\right)<\delta {\mathrm{Q̅}}_{i}^{n}\\ {\mathrm{Q̅}}_{i}^{n}\left(\Gamma_{j-1}\right) & \mathrm{otherwise} \end{array}$$

and $k_{i}^{n}\left(\Gamma_{j}\right)$

12 $$k_{i}^{n}\left(\Gamma_{j}\right)=\{\begin{array}{ll} k_{i}^{n}\left(\Gamma_{1}\right) & \delta \Lambda_{i}^{n}\left(\Gamma_{j-1}\right)>\delta {\mathrm{Q̅}}_{i}^{n}\\ k_{i}^{n}\left(\Gamma_{j-1}\right)/C_{dk}\left(i\right) & 0<\delta \Lambda_{i}^{n}\left(\Gamma_{j-1}\right)<\delta {\mathrm{Q̅}}_{i}^{n}\\ k_{i}^{n}\left(\Gamma_{j-1}\right)\cdot C_{dk}\left(i\right) & \mathrm{otherwise} \end{array}$$

with

13 $$\delta \Lambda_{i}^{n}\left(\Gamma_{j-1}\right)=\left[Q_{i}^{\mathrm{ex}}\left(\Gamma_{j-1}\right)-{\mathrm{Q̅}}_{i}^{n}\left(\Gamma_{j-1}\right)\right]\cdot u_{i}^{n}$$

where $u_{i}^{n}$ is given by

14 $$u_{i}^{n}=\frac{\delta {\mathrm{Q̅}}_{i}^{n}}{\left|\delta {\mathrm{Q̅}}_{i}^{n}\right|}$$

and

15 $$Q_{i}^{\mathrm{ex}}\left(\Gamma_{j-1}\right)=\underset{0<\tau <t}{\max}\left(Q_{i}\left(\tau \right)\cdot u_{i}^{n}\right)$$

### 2.3 Conformer Recognition, Geometry Optimization, and Spectrum Calculation

For the geometry optimization and subsequent spectral calculations in step 5, high-level DFT methods (hybrid functionals and sufficiently polarized basis sets) are required to achieve accurate spectra.^40–47^ However, the biased MD of step 3 only needs to provide reasonable structures, i.e., it only needs to approximately locate the minima on the PES. This can be achieved with lower-level theories—such as GGA functionals or even tight-binding methods—for many systems.

To reduce the overall computational cost without compromising accuracy, different levels of theory are used for steps 3 and 5. Moreover, because step 5 (geometry optimization and the corresponding spectrum calculation) is the most expensive and time-consuming step of SPICES, to avoid unnecessary and redundant calculations, a stepwise geometry optimization and conformer recognition procedure was implemented. When a new minimum region is identified in the MD, by analyzing the temporal evolution of energy (see Supporting Information Section S2 for more details), the corresponding structure ***Q*** ^*X*^ is compared to three reference sets: (i) the already tested MD structures, {***Q*** ^ref^}_MD_; (ii) previously optimized structures at the same level of theory as the MD, {***Q*** ^ref^}_MD–OPT_; and (iii) the set of structures reoptimized at a higher level, {***Q*** ^ref^}_HIGH–OPT_. If the new structure is significantly different from these reference structures by geometrical criteria (see Section 3.3 for more details), a preoptimization is performed at the same level of theory as the MD. The resulting geometry is checked again against {***Q*** ^ref^}_MD–OPT_ and {***Q*** ^ref^}_HIGH–OPT_. If this second screening is also passed, the geometry is optimized at a high level. Finally, the fully optimized geometry is checked one last time by comparing it against the {***Q*** ^ref^}_HIGH–OPT_ set. Only conformations that successfully pass all tests are regarded as genuinely new, and their spectra are subsequently computed. The newly obtained reference structures are added to the three reference lists. If, instead, a check fails at any point, the biased MD continues searching for another candidate structure.

### 2.4 Spectral Match and LCCS Score

For the required quantitative comparison of spectra (step 6), we selected the Logarithmic Convoluted Cosine Similarity (LCCS) score, introduced in our previous work^42^ to which we refer for a detailed discussion of this metric and its advantages over other scores proposed in the literature. Here, in brief, the LCCS score between two spectra $I_{0}^{A}$ and $I_{0}^{B}$ is defined as

16 $$LCCS\left(A,B\right)=\frac{I^{A}F\left(\lambda \right)F\left(\lambda \right){\left(I^{B}\right)}^{T}}{\left|I^{A}F\left(\lambda \right)\right|\left|F\left(\lambda \right){\left(I^{B}\right)}^{T}\right|}$$

where ***I*** ^*X*^ (with X equal to either A or B) is a vector for which the *i*-th element, *I* ^*X*^(*i*), is defined as

17 $$I^{X}\left(i\right)=\mathrm{sgn}\left(I_{0}^{X}\left(i\right)\right)\frac{\log\left(c\;|I_{0}^{X}\left(i\right)|+1\right)}{\log\left(c+1\right)}$$

Here, the spectrum $I_{0}^{X}$ is scaled and discretized using the following protocol. As a first step, the spectrum *X* is scaled so that the maximum intensity is set to 1. The frequency range is then partitioned using a regular grid with a spacing of Δ, and the intensity of *X* corresponding to each grid point is stored in the vector $I_{0}^{X}$ . For an experimental spectrum, the data spacing is dictated by the characteristics of the measuring instrument. However, e.g., a cubic spline interpolation can be used to achieve the desired spacing. *c* is an empirical factor that can be adjusted to either emphasize the frequency shifts (larger *c*) or intensity mismatches (smaller *c*). **F** in eq 16 is the fold matrix,^70^ with elements

18 $$F_{ij}\left(\lambda \right)=\frac{1}{1+{\left(\frac{2\Delta }{\lambda }\right)}^{2}{\left(i-j\right)}^{2}}$$

where λ is the full width at half-maximum (fwhm) and Δ is the grid spacing of the intensities. The LCCS score ranges from 0 to 1 in the case where the spectra are only positively defined, e.g., IR and Raman spectra, and between −1 and 1 if the spectra can also assume negative values, e.g., in the case of Vibrational Circular Dichroism (VCD). The higher the LCCS score, the better the matching between the two spectra, with one corresponding to a perfect match and zero corresponding to no match at all. Compared to other scores proposed in the literature, LCCS is not defined solely by the most intense peaks but is also sensitive to the smaller ones. Even more importantly, LCCS differentiates between small frequency mismatches and significant band shifts.

When comparing computed and experimental spectra, small shifts in band positions are expected due to inherent limitations of the theoretical models and unavoidable experimental noise. Consequently, a perfect match is not anticipated, and the LCCS value is never exactly equal to 1, even for the correct structure. In SPICES, the comparison is therefore considered satisfactory—i.e., a solution is deemed to be found—when the LCCS score exceeds an acceptance threshold. This threshold is a method parameter that must be adjusted according to the noise level of the target spectrum and the level of theory employed.

### 2.5 Dimensionality Reduction of the Spectrum

A standard intensity vector ***I*** ^ref^ consists of thousands of elements. These are too many to use directly as input to any numerical method for building ***s***_*n*_ as it will quickly lead to overfitting. A dimensionality reduction protocol is therefore applied. The frequency range is partitioned into bins of fixed width without a stride. The total cumulative intensity within each bin width is computed and assigned to that bin. The width is carefully chosen. Increasing the bin width reduces the total number of bins, thereby improving the efficiency of dimensionality reduction. However, this also results in a lower spectral resolution, increasing the risk of deletion of relevant features. The binned spectrum is first normalized so that its maximum intensity equals 1. The full set of binned spectra is then standardized, with each bin having zero mean and unit variance. As a last step, principal component analysis (PCA) is performed on the binned spectra. Starting with the first, the principal components are selected until 99% of the variance in the data is accounted for. This provides a new set of features that is significantly smaller than the initial set but that still contains the relevant information.

### 2.6 Frequency Scaling Factor

As already noted in the previous section, discrepancies between theoretical spectra and experimental results are expected because of anharmonic effects, incomplete basis sets, and inadequate treatment of electron correlation. In general, improving the level of theory reduces these errors, although such improvements typically entail an increased computational cost. A common strategy to contain costs is to use a lower level of theory, e.g., by avoiding expensive anharmonic calculations and compensating for systematic errors with scaling factors to correct the computed frequencies. This was also implemented here.

Because the optimal scaling factor for a system is not always known *a priori*, a strategy that optimizes it during SPICES for a specific system under study has been designed. For each ***I*** ^ref^, the scaling factor is optimized via a grid search to maximize the LCCS score, i.e., the spectral similarity, and is integrated into the procedure at step 6. In this way, each ***Q*** ^ref^ is given the best opportunity to match the experimental spectrum.

As a sanity check for this protocol, we compared the results obtained with this adaptive approach to those from a fixed-scaling-factor strategy, using either standard tabulated scaling factors from the CCCBDB database^71^ or scaling factors specifically optimized for the benchmark system. These results are reported in Supporting Information (Section S3). Here, we limit ourselves to the observation that a fixed-scaling-factor strategy provides the best performance only when the chosen scaling factor is optimal. However, as soon as this condition is not met—for example, in the case of our benchmark molecule, where the optimal scaling factor is 1.001 for PBE-D3­(BJ)/6-31G­(d,p) while the CCCBDB value is 0.986—the adaptive optimization strategy clearly outperforms the fixed approach.

### 2.7 History-Dependent Displacement Field

One of the key ingredients of the method presented here is the history-dependent displacement field ***s***_*n*_(***I*** *, ***I*** ^*n*^, ***Q*** ^*n*^|{***I*** ^ref^, ***Q*** ^ref^}), which is determined in step 2 and used in step 3 to inform the bias. For the method to be efficient, ***s***_*n*_ must be easy and cheap to evaluate on the fly. Moreover, because SPICES starts without any preliminary knowledge, ***s***_*n*_ must provide a reasonable guess even when few reference structures (fewer than 20) are known. For this reason, we selected a linear shape:

19 $$s_{n}\left(I^{n},I^{*},Q^{n}\right)=B^{T}{Ĩ}^{*}-Q^{n}$$

where the vector ${Ĩ}^{*}=\left[1I_{1}^{*}\;I_{2}^{*}\;I_{3}^{*}...\right]$ is the augmented form of ***I*** * and ***B*** ^*T*^ is the transpose of ***B***. We tested several options for defining the matrix ***B***. In the first case, ***B*** is defined as a function of the {***I*** ^ref^, ***Q*** ^ref^} set, i.e., ***B*** = ***B*** ({***I*** ^ref^, ***Q*** ^ref^}) and obtained via simple linear regression. Let ***X*** be the matrix that collects the augmented forms of {***I*** ^ref^},

20 $$X=\left[1|\left\{I^{\mathrm{ref}}\right\}\right]=\left[\begin{array}{llll} 1 & I_{1}^{1} & \cdots & I_{m}^{1}\\ 1 & I_{1}^{2} & \cdots & I_{m}^{2}\\ \vdots & \vdots & \ddots & \vdots \\ 1 & I_{1}^{n} & \cdots & I_{m}^{n} \end{array}\right]$$

Let ***Y*** be the matrix containing the corresponding coordinates {***Q*** ^ref^}; then, the coefficient matrix is given by

21 $$B={\left(X^{T}X\right)}^{-1}X^{T}Y$$

This was calculated using the implementation of scikit-learn.^72^ This purely linear strategy will be called SPICES_LR_.

However, the space we are dealing with is inherently nonlinear, and the assumed linear form might be overly crude or simplistic. One possible way to address this limitation would be to use a higher-order polynomial instead. Such an approach, however, would require substantially more data to obtain a reliable fit—data that are not available during the initial stages of SPICES. Therefore, an alternative strategy is tested here, where we use a weighted linear fit. The underlying idea of the strategy is that locally the space is still rather linear and hence that a linear model can be predictive if it is predominantly based on close-lying structures. The weights in our new locally linear model follow a Gaussian distribution. If the set of {*q*_*i*_} is homogeneous, it is possible to introduce a metric *d*_αβ_ = | ***R*** ^α^ – ***R*** ^β^| that measures the distance between two system structures (α and β) such that

22 $$w_{\alpha \beta }=\frac{\exp\left(-\frac{d_{\alpha \beta }^{2}}{2\sigma^{2}}\right)}{\underset{j}{\sum }\exp\left(-\frac{d_{\alpha j}^{2}}{2\sigma^{2}}\right)}$$

If {*q*_*i*_} is not a homogeneous set, the equation can be generalized to

23 $$w_{\alpha \beta }=\frac{\underset{x}{\prod }w_{\alpha \beta }^{x}}{\underset{j}{\sum }\underset{x}{\prod }w_{\alpha j}^{x}}$$

where *x* represents each homogeneous subset. The history-dependent displacement field ***s***_*n*_ can then be obtained by rewriting eqs 19 and 21 with a new augmented matrix ***X***_*n*_ that collects the {*w*_*n*,ref_ · ***I*** ^ref^} elements and a new matrix ***Y***_*n*_ that collects the corresponding {*w*_*n*,ref_ · ***Q*** ^ref^},

24 $$s_{n}\left(I^{n},I^{*},Q^{n}\right)=B_{n}^{T}{Ĩ}^{*}-Q^{n}$$

with

25 $$B_{n}={\left(X_{n}^{T}X_{n}\right)}^{-1}X_{n}^{T}Y_{n}$$

This strategy will be called SPICES_LR–W_ in the discussion section. Notice that in this case, ***B***_*n*_ depends parametrically on ***Q*** ^*n*^, i.e., ***B*** = ***B*** ({***I*** ^ref^, ***Q*** ^ref^}| ***Q*** ^*n*^). Incorporating the weight factor into the model using standard weighted linear regression techniques was also evaluated. However, this approach yielded an inferior performance compared to the strategy proposed here and is therefore not discussed further; details are provided in the Supporting Information (Section S5.5). Finally, we also tested a strategy in which the weight is computed from the distance in the spectral space, measured by the LCCS score (SPICES_LR–LCCS_), rather than from the structural parameters. No performance improvements from using LCCS-based weights were observed. Therefore, this approach is not discussed further here, but the corresponding results are still reported in the Supporting Information (Section S5.4).

### 2.8 Initial Structure Generation

At the start of SPICES, only one structure and spectrum are available, which is not enough to build the history-dependent displacement field in step 2. Therefore, a special protocol for the first iteration must be devised. Two strategies were tested: SPICES^R^ and SPICES^HT^. SPICES^R^ uses random biases for the first iteration. In the second iteration, two reference structures and spectra are available; therefore, ***s***_*n*_ can be obtained in the standard way.

However, the number of reference structures and spectra during the first couple of iterations of the SPICES^R^ strategy remains limited, making ***s***_*n*_ unreliable for predicting good moves. Therefore, the second strategy (SPICES^HT^) introduces a pregeneration of structures, providing an initial set of references for the first iteration of SPICES. This pregeneration entails a 15 ps unbiased molecular dynamics simulation at high temperature. Because of the high temperature, several stable conformers will be explored. These are recognized, further optimized, and used for spectrum calculations, thereby building a sparse initial reference set {***I*** ^ref^, ***Q*** ^ref^}. This set is then used to initialize ***s***_1_. Within this strategy, it is then possible to choose any of the structures in {***Q*** ^ref^} as a starting point. Here, the choice is to use the structure with the spectrum that has the largest LCCS value relative to the target spectrum. Other strategies were also investigated and are critically compared in the Supporting Information (Section S5.2).

### 2.9 Summary

In summary, the SPICES workflow to predict the conformer(s) corresponding to the target spectrum ***I*** * consists of the following steps:

0. The procedure starts by providing a random initial structure and selecting the set of coordinates {*q*_i_} whose values {*Q*_i_} are used in SPICES.

1. SPICES is initialized using either the SPICES^R^ or SPICES^HT^ strategy (see Section 2.8).

From this point onward, the iterative procedure of SPICES begins. At the *n* ^th^ iteration:

2. The history-dependent displacement field ***s***_*n*_ is fitted to the previously explored *n* structures, {***I*** ^ref^, ***Q*** ^ref^}, either using the SPICES_LR_ or SPICES_LR–W_ strategy (see Section 2.7 for more details).

3. Based on ***s***_*n*_, the initial values of the adaptively biased potential at the *n* ^th^ SPICES iteration, *V* ^*n*^(*t*, ***Q***), are set (see Section 2.2)

4. The biased MD is run until a possible new minimum is found (see Section 2.3).

5. The obtained structure is optimized, and the spectrum is computed, obtaining ***Q*** ^n+1^ and ***I*** ^n+1^.

6. The LCCS­(***I*** ^n+1^, ***I*** *) score is computed (see Section 2.4).

- If the LCCS score is above the acceptance threshold, then a solution to the problem has been found.
- If the comparison is not satisfactory, ***Q*** ^n+1^ and ***I*** ^n+1^ are added to the {***I*** ^ref^, ***Q*** ^ref^} set, and a new iteration starts (step 2).

If multiple 3D structures are expected to correspond to the target spectrum ***I*** *, then SPICES is run again, looking for the other solutions.

## 3 Computational Details

### 3.1 Test System

As a test system, we selected gas-phase diphenylalanine (FF)^73^ (Figure 2). This molecule is of particular scientific interest because it represents the simplest peptide building block capable of self-assembling into ordered nanostructures whose topology can also be modulated by environmental conditions, thereby making this system highly attractive for technological applications.^74,75^ Additionally, FF is a key structural motif in β-amyloid^76,77^ and, as such, has been studied in relation to Alzheimer’s disease and other neurodegenerative diseases.

**Figure 2:**
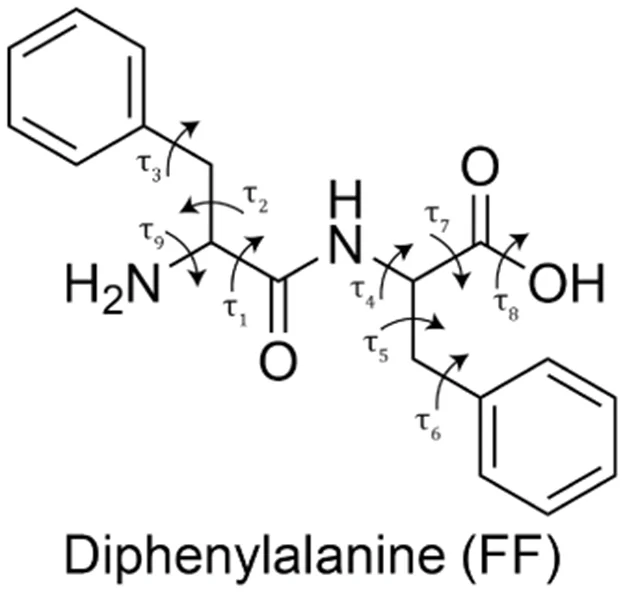
Molecular structure of Diphenylalanine (FF) with the arrows indicating the torsional angles used for {*q_i_*}.

For SPICES, we select the nine dihedral angles indicated by arrows in Figure 2 as the set of coordinates {*q*_*i*_}, whose values {*Q*_*i*_} uniquely identify the system structure. Cyclic periodic boundary conditions and symmetry are taken into account in building ***s***_*n*_ and in recognizing the minima of the PES; see Supporting Information (Section S1) for more details.

For the ratchet-and-pawl-like potential parameters (***C*** ^*r*^, ***C*** ^*k*^, and ***C***_*dk*_; eqs 8,9,12), we used the same values for all nine torsional angles; the values are reported in Table 1. In computing the LCCS score, the convolution width has been fixed to 5 cm^–1^, and *c* was set to 5. For the binning procedure used in the dimensionality reduction protocol (see Section 2.5), a bin size of 5 cm^–1^ was selected. Finally, for SPICES_LR–W_, σ^2^ was fixed to 5 rad^2^.

**Table 1:** Parameters Used to Set Up and Dynamically Adjust the Asymmetric Ratchet-and-Pawl Like Potential *V^n^* (*t*, ***Q***); The Same Values Have Been Used for All Nine Torsional Angles of FF

Length of the macrostep	500 MD steps
***C*** ^*r*^	$\frac{\pi }{10}\mathrm{rad}$
***C*** ^*k*^	0.075 hartree/rad
***C***_*dk*_	1.1

### 3.2 MD Simulations

All biased molecular dynamics simulations reported in this work were performed using the CP2K code,^78^ coupled with PLUMED^79,80^ to apply the biasing potential. Born–Oppenheimer molecular dynamics were used, i.e., at each time step, the electronic wave function was converged, and the forces were computed, imposing a threshold for the energy difference between two SCF cycles of 10^–7^ Hartree/cell. The classical Newton’s equations of motion for the nuclei were integrated through the velocity Verlet algorithm with a time step of 0.5 fs. In our previous work,^42^ we showed that for systems such as the present test case, semiempirical methods, such as DFTB3 with the 3-ob-3–1 parametrization^81^ and the D3­(BJ) dispersion correction,^82,83^ are sufficiently accurate for a preliminary map of the potential energy surface (PES). They are therefore the methods of choice in the present study during the MD simulations.

The simulation box dimensions were chosen to be 20 Å × 20 Å × 20 Å to ensure negligible interaction between periodic replicas. The trajectories were run in the *NVT* ensemble. The system average temperature was set to 300 K, and a CSVR thermostat^84^ was applied (time constant 25 fs), with the velocities automatically rescaled whenever the temperature fluctuations exceeded 300 K ± 100 K. For the special case of the initial high-temperature sampling step (i.e., the initialization stage of SPICES^HT^), the temperature was increased to 600 K. This simulation was run for 15 ps.

### 3.3 Geometry Optimizations and Spectral Calculations

To minimize the number of high-level calculations, the stepwise geometry optimization and conformer recognition procedure described in section 2.3 is used. We consider a conformer significantly different if any of the tracked coordinates shows a larger difference than the cutoff values summarized in Table 2. Since the selected set {*q*_*i*_} consists exclusively of torsional angles, the same cutoff was applied to all coordinates. The preoptimization is done with the CP2K code, using DFTB3 with the 3-ob-3-1 parametrization and D3­(BJ) dispersion corrections, i.e., the same level of theory used for the biased MD itself. The convergence criteria were set to 3 × 10^–3^ bohr (maximum) and 1.5 × 10^–3^ bohr (RMS) for the change in atomic positions between optimization steps, and to 4.5 × 10^–4^ Hartree bohr^–1^ (maximum) and 3 × 10^–4^ Hartree bohr^–1^ (RMS) for the corresponding forces. The used SCF cutoff was set to 10^–7^ Hartree.

**Table 2:**
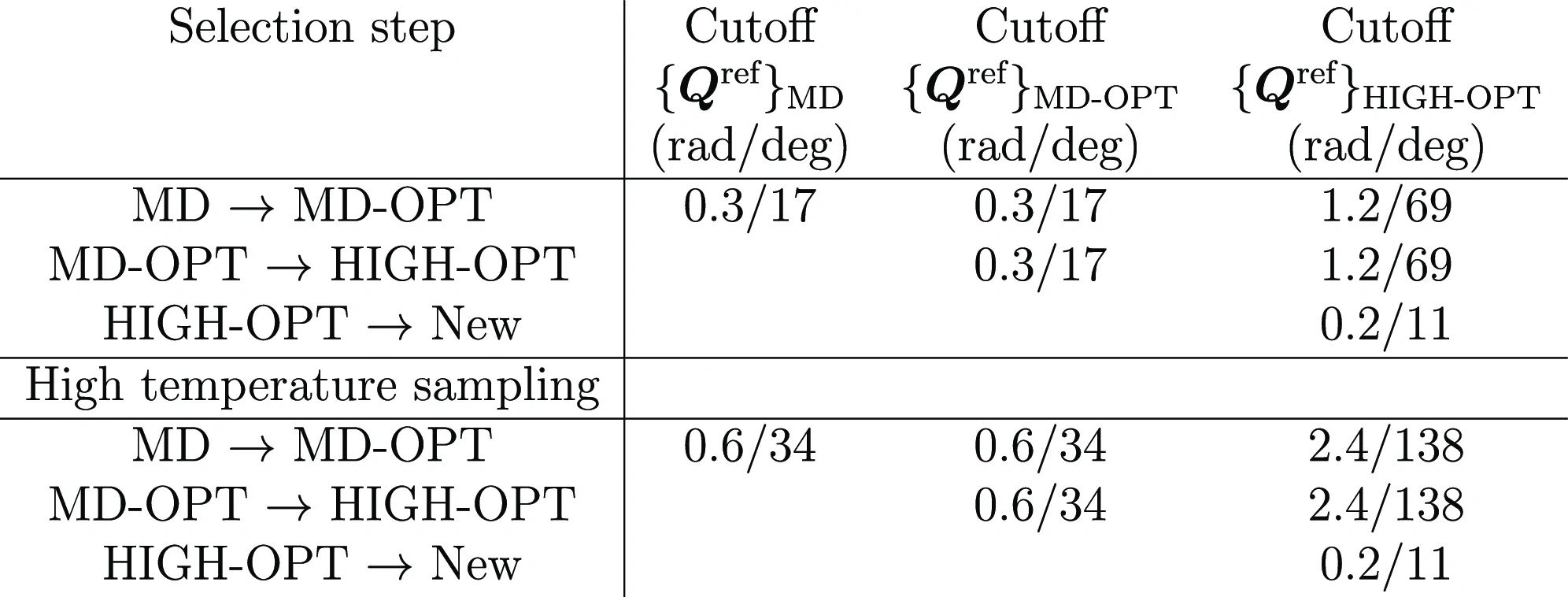
Cutoff Values Used to Determine if a Structure Differs Enough from the Other Structures in the Reference Set to Pass to the Next Step in the Multilevel Geometry Optimization Procedure

Subsequently, the structure is reoptimized with a higher level of theory, and the spectra are calculated. For all results discussed in this paper, the Gaussian16 code^85^ was used for this step. We tested the performance of the method both using the PBE^86^ functional with the D3­(BJ) dispersion corrections^82,83^ in combination with the 6-31G­(d,p) basis set and the B3LYP functional^87,88^ with the D3­(BJ) dispersion corrections and the 6-311G­(d,p) basis set. The geometry is fully optimized with tight cutoff values for the convergence, i.e., 3 × 10^–3^ bohr (maximum) and 1.5 × 10^–3^ bohr (RMS) for the change in atomic positions between optimization steps, and 4.5 × 10^–4^ Hartree bohr^–1^ (maximum) and 3 × 10^–4^ Hartree bohr^–1^ (RMS) for the corresponding forces. The used SCF cutoff was the default value of 10^–8^ Hartree. Finally, the spectra have been obtained for these fully optimized geometries in the double-harmonic approximation. The same SCF cutoff of 10^–8^ was used. The obtained stick spectrum is convoluted with a Lorentzian of width 10 cm^–1^.

## 4 Results and Discussion

Figure 3 provides an example of a typical SPICES run. The top panel shows the evolution of the LCCS score. The central panel tracks the evolution of the torsional angles relative to those of the target structure. The bottom panel compares the computed spectrum (red line) with the target experimental spectrum (brown line). The initial spectrum (Iteration 0) exhibits significant discrepancies with respect to the target experimental spectrum, as highlighted by the difference spectrum (pink line). For example, the carboxylic acid C=O stretching band at 1700 cm^–1^ is clearly shifted relative to the target spectrum. This mismatch originates from the presence of a hydrogen bond between the COOH group and the C=O group of the amide in the initial structure, which is absent in the target structure, as indicated by Δτ_8_. After approximately 15 iterations, the spectral region below 1600 cm^–1^ begins to converge toward the target spectrum, whereas the higher-frequency region requires additional iterations to achieve a comparable level of agreement. This behavior reflects the fact that some torsional angles (e.g., τ_3_ and τ_6_) are easier to find than others. In particular, τ_9_ oscillates up to the final match. In the following, we will discuss the performance of different strategies and modules in SPICES. To better assess the performance of the different SPICES configurations in the final results and to enable a more comprehensive comparison among the various configurations, we first focus exclusively on the history-dependent displacement field and its initialization (Section 4.1). The MD component is then introduced into SPICES (Section 4.2). To simplify notation, in the following discussion, PBE-D3­(BJ)/6-31G­(d,p) will be abbreviated to PBE, and B3LYP-D3­(BJ)/6-311G­(d,p) to B3LYP.

**Figure 3:**
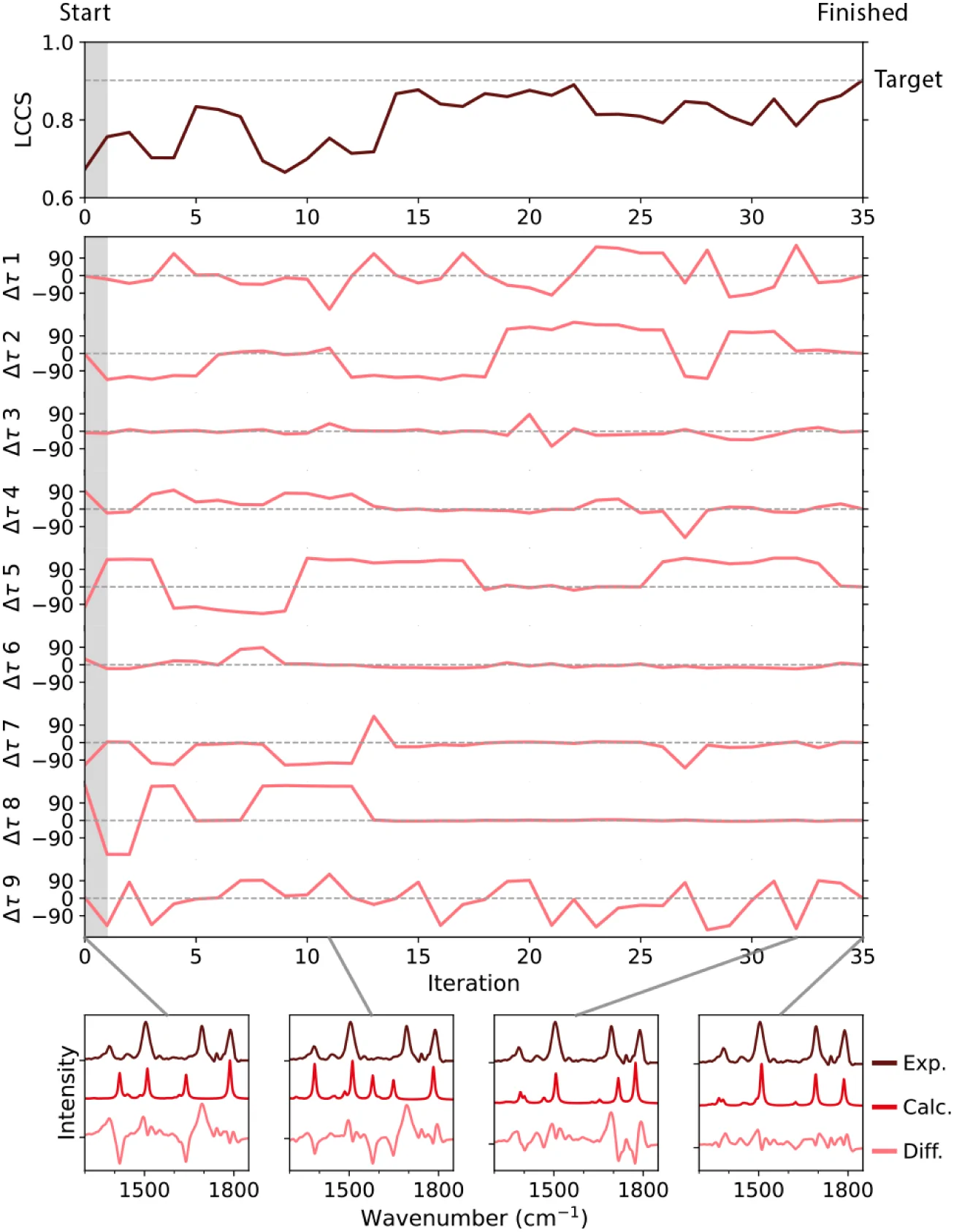
Overview over how a typical run in the ${\mathrm{SPICES}}_{\mathrm{LR‐W}}^{\mathrm{HT}}$ configuration evolves over time. Top panel: LCCS score. Central panel: evolution of the torsional angles relative to those of the target structure. Bottom panels: comparison of the computed spectrum at each iteration (red line) with the target experimental spectrum (brown line). The pink line shows the difference spectrum. The gray background indicates the high-temperature sampling steps.

### 4.1 History-Dependent Displacement Field and Its Initialization

To evaluate the selected history-dependent displacement field ***s***_*n*_ (SPICES_LR_ and SPICES_LR–W_) and the initialization strategy (SPICES^R^ and SPICES^HT^) independently of the MD performance, the MD component of SPICES is replaced by an artificial exploration of the conformational space (A-SPICES). Here, we make use of a data set {***I*** ^full^, ***Q*** ^full^} comprising 404 distinct conformations of the FF molecule, obtained from an extensive PES scan. This data set is then used as a controlled playground in which the MD step of SPICES is substituted by a walker that jumps directly between PES minima. The exact rules for this jumping are outlined in the Supporting Information (Section S4).

Initially, the performance of the different A-SPICES configurations is assessed using computed spectra as targets. This is done to exclude negative effects arising from noise in an experimental spectrum and from mismatches between theory and experiment. The selected spectral range is from 1 to 4000 cm^–1^, with a spacing (Δ) of 1 cm^–1^. Five targets have been randomly selected from {***I*** ^full^} and 50 random starting points from {***Q*** ^full^}. The target set includes both low- and high-energy conformers, since one goal of the method is to remain effective even when the target is not the global minimum and, more generally, to operate independently of energy-based criteria. For each combination of starting point and target, A-SPICES is run 50 times, resulting in a total of 12500 runs. To critically compare the method configurations ( $A‐{\mathrm{SPICES}}_{\mathrm{LR}}^{R}$ , $A‐{\mathrm{SPICES}}_{\mathrm{LR}}^{\mathrm{HT}}$ , $A‐{\mathrm{SPICES}}_{\mathrm{LR‐W}}^{R}$ , and $A‐{\mathrm{SPICES}}_{\mathrm{LR‐W}}^{\mathrm{HT}}$ ), two complementary metrics have been considered. The first is the *X*-success-rate (*X*-S-R), defined as the percentage of trajectories that find a solution within *X* unique explored conformers, i.e., *X* high-level DFT calculations. Here, we selected X = 100, which represents a reasonable upper limit for the number of harmonic spectrum calculations one would typically like to perform for a medium-sized molecule. The second metric is the median of the number of unique explored conformers required to find a solution. We use the median rather than the mean to reduce the influence of outliers and unfinished trajectories. Trajectories that did not finish within the 100 criterion were included by assigning them a value of 101. Both metrics are based on the number of unique explored conformers because the high-level calculations (geometry optimization and frequency calculation) performed for each new conformer account for most of the computational cost in SPICES and therefore provide the best proxy for the overall computational cost.

In Figure 4, the values obtained for the test system using the PBE level of theory are reported. The trends observed at the B3LYP level of theory are similar and are therefore not discussed here, but they can be found in the Supporting Information (Section S5). Compared to the results obtained with a random walk between the minima (RANDOM), the basic configuration of A-SPICES, employing a simple linear regression and no initial presampling $\left(A‐{\mathrm{SPICES}}_{\mathrm{LR}}^{R}\right)$ , already yields excellent results (Figure 4). The median decreases from 223 unique found conformers to only 49, and the 100-S-R increases from 23.1% to 71.6%. Both the probability-weighted linear regression $\left(A‐{\mathrm{SPICES}}_{\mathrm{LR‐W}}^{R}\right)$ and the initial high-temperature sampling $\left(A‐{\mathrm{SPICES}}_{\mathrm{LR}}^{\mathrm{HT}}\right)$ strategies further improve performance. When either strategy is used individually, the median decreases to 31 and the 100-S-R increases above 90%. The best performance is achieved when the probability-weighted linear regression and initial high-temperature sampling strategies are combined $\left(A‐{\mathrm{SPICES}}_{\mathrm{LR‐W}}^{\mathrm{HT}}\right)$ : a median of only 28 and a 100-S-R of 98%. On the basis of these results, we conclude that the ***s***_*n*_ benefits from adding some nonlinearity and from an initial sampling of the conformational space. Additional strategies, including a Monte Carlo approach for move acceptance and an alternative method for computing probability-based weights based on the LCCS score, were also tested. Since these strategies do not yield clear improvements, they are not discussed here; however, the results are reported in the Supporting Information (Section S5).

**Figure 4:**
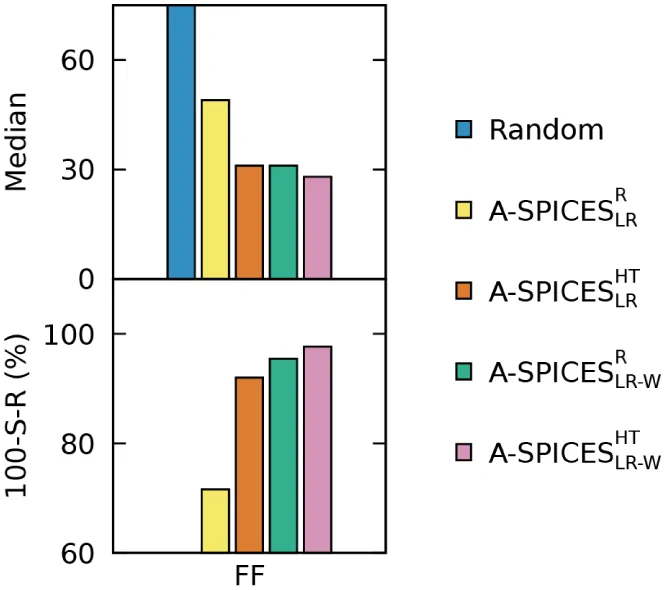
Comparison of the performance of different A-SPICES configurations (see Section 2 for definitions). Performance is evaluated over 12500 runs, comprising 5 random targets from {***I*** ^full^} and 50 random starting points from {***Q*** ^full^}, with each start–target pair repeated 50 times. Performance is assessed using (i) the median number of unique found conformers required to find a solution and (ii) the 100-S-R, defined as the percentage of trajectories that found a solution in fewer than 100 unique found conformers. Results obtained using a random walk (median equal to 223, 100-S-R 23.1%) are also included for comparison.

As a second step, the A-SPICES performance, in particular in its $A‐{\mathrm{SPICES}}_{\mathrm{LR‐W}}^{\mathrm{HT}}$ configuration, is critically assessed against an experimental spectrum. The specific experimental target spectrum is the gas-phase spectrum of FF as reported in ref 73 (from 290 to 2000 cm^–1^ spectral range). The spectrum was measured using IR–UV ion-dip spectroscopy, which ensures that only a single conformer contributes to the experimental infrared spectrum, making it ideal for benchmarking the implemented method. In particular, the experimental spectrum can be assigned to the MP2/6-311++G­(d,p) global minimum conformation.^42,73^ For this test, the B3LYP level of theory, which we have previously shown^42^ to provide high-quality IR spectra for the FF molecule, was selected.

As explained in Section 2.4, when an experimental spectrum is used as a target, a perfect match cannot be expected, even when high-quality spectra are employed; therefore, an acceptance threshold lower than 1 must be adopted. In particular, we selected a threshold of 0.902 to ensure that only one possible match exists in our data set. Again, 50 random starting points from {***Q*** ^full^} were selected, and $A‐{\mathrm{SPICES}}_{\mathrm{LR‐W}}^{\mathrm{HT}}$ is run 50 times for each, resulting in a total of 2500 runs. The results, reported in Figure 5, show that $A‐{\mathrm{SPICES}}_{\mathrm{LR‐W}}^{\mathrm{HT}}$ efficiently provides the correct assignment of the experimental spectrum with a median number of 32 unique conformers found and a 100-S-R of 98.6%. Indeed, the chosen ***s***_*n*_ definition and the optimization strategy for the frequency scaling factor (see Section 2.2) demonstrate robustness to noise and small mismatches between theory and experiment. The situation differs when the discrepancy becomes large. Indeed, when a lower-quality method, still suitable for qualitative comparisons but not for quantitative ones, is employed (in our case, PBE), a significantly poorer performance is observed, with a median number of 75 unique conformers found and a 100-S-R below 70%. The difficulty of PBE in correctly describing the spectral differences between conformers seems to strongly hinder SPICES. For further details, see Supporting Information (Section S3).

**Figure 5:**
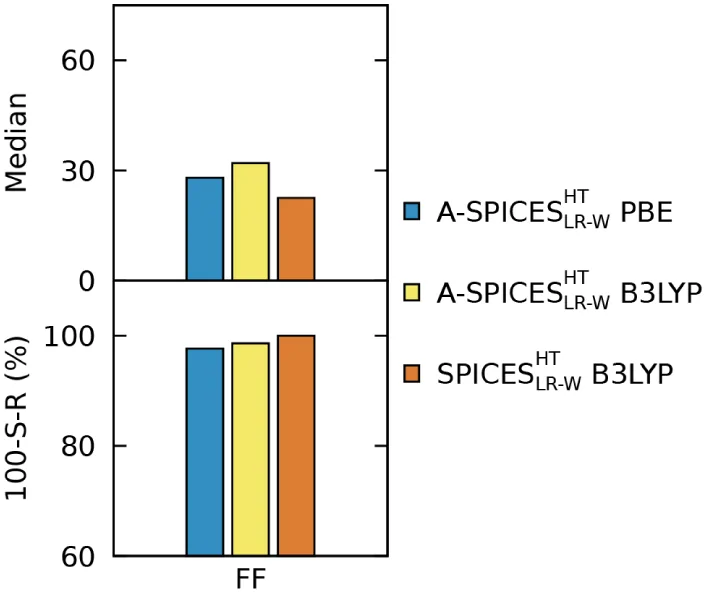
Comparison of the performance of $A‐{\mathrm{SPICES}}_{\mathrm{LR‐W}}^{\mathrm{HT}}$ and ${\mathrm{SPICES}}_{\mathrm{LR‐W}}^{\mathrm{HT}}$ using the experimental spectrum as the target. The spectra have been computed in double-harmonic approximation at either the PBE or the B3LYP level. For $A‐{\mathrm{SPICES}}_{\mathrm{LR‐W}}^{\mathrm{HT}}$ , the performance is evaluated over 2500 runs, comprising 50 random starting points from {***Q*** ^full^}, repeated 50 times. For ${\mathrm{SPICES}}_{\mathrm{LR‐W}}^{\mathrm{HT}}$ , the same 50 random starting points were used, but no repetitions were performed due to computational costs, resulting in 50 runs. In all cases, the performance is assessed using (i) the median number of unique found conformers required to find a solution and (ii) the 100-S-R, defined as the percentage of trajectories that found a solution in fewer than 100 unique found conformers.

Finally, to assess SPICES’s ability to sample multiple solutions, an additional test was carried out at the B3LYP level by lowering the acceptance threshold for matching the experimental spectrum to 0.893. In this case, three candidate matches for the experimental spectrum were present in the database. SPICES identifies the conformer with the highest LCCS score in 40% of the runs, whereas the remaining two conformers are found in 28% and 32% of the runs, respectively (see Supporting Information Section S6 for more details), thereby confirming that SPICES has the ability to sample different solutions.

### 4.2 SPICES with Biased-MD

Having critically assessed the performance of the history-dependent displacement field and the initialization strategy, we now evaluate SPICES performance when the real, biased molecular dynamics simulation is used to explore the conformational space and identify the next minimum on the fly. As in the previous section, the behavior of SPICES is first tested using computed spectra as targets to exclude effects arising from noise in an experimental spectrum and from mismatches between theory and experiment. The selected spectral range is from 1 to 4000 cm^–1^, with a spacing (Δ) of 1 cm^–1^. Because a regular SPICES trajectory (with the MD) is significantly more computationally expensive than an A-SPICES run, this analysis has been performed at the PBE level of theory for the spectra, and fewer runs have been performed than in the previous section. We select 50 starting conformers, but consider only two target structures. For each start–target pair, SPICES is performed only once. The two targets have been carefully chosen to be a low-energy and a high-energy conformer, to test again SPICES’ ability to be independent of energy criteria in finding the solution. The low-energy target is the global minimum on the MP2/6-311++G(d,p) surface. Note that, although this conformer remains a stable minimum on the PBE surface, it is no longer the lowest-energy structure, lying 5.2 kJ/mol above the global minimum at the PBE level of theory. The higher-energy target lies 26.5 kJ/mol above the PBE level of theory global minimum, placing it well outside the energy window of conformers favored by a Boltzmann distribution at room temperature. Because the tests reported in Section 4.1 demonstrated that the weighted linear regression (LR-W) combined with the initial high-temperature sampling (HT) configuration performs best, we adopt this setup here. Figure 6 reports the results obtained with ${\mathrm{SPICES}}_{\mathrm{LR‐W}}^{\mathrm{HT}}$ , together with the corresponding $A‐{\mathrm{SPICES}}_{\mathrm{LR‐W}}^{\mathrm{HT}}$ results. For ${\mathrm{SPICES}}_{\mathrm{LR‐W}}^{\mathrm{HT}}$ , the results are further split between the low-energy and high-energy targets. In general, ${\mathrm{SPICES}}_{\mathrm{LR‐W}}^{\mathrm{HT}}$ shows slightly lower performance than $A‐{\mathrm{SPICES}}_{\mathrm{LR‐W}}^{\mathrm{HT}}$ . For the combined (i.e., low-energy + high-energy target) results, the median is 10 higher, and the 100-S-R is around 10% lower. Nonetheless, ${\mathrm{SPICES}}_{\mathrm{LR‐W}}^{\mathrm{HT}}$ performance (median of 37 unique conformers, and 100-S-R is approximately 90%) can still be considered excellent, especially in comparison to other data-driven approaches that required large training sets (>200 spectra).^34,57^

**Figure 6:**
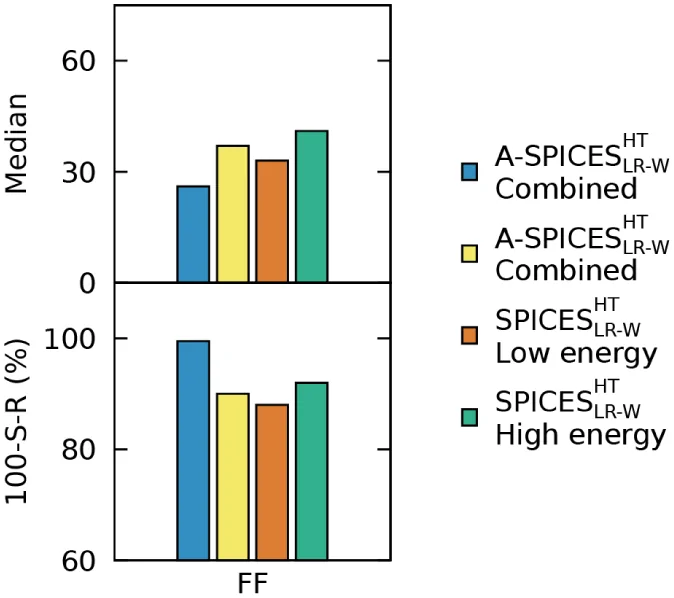
Comparison of the performance of $A‐{\mathrm{SPICES}}_{\mathrm{LR‐W}}^{\mathrm{HT}}$ and ${\mathrm{SPICES}}_{\mathrm{LR‐W}}^{\mathrm{HT}}$ using as the target a low-energy or a high-energy conformer. The combination of the two is also reported for reference. For A-SPICES^HT^LR-W, the performance is evaluated over 2500 runs, comprising 50 random starting points from {***Q*** ^full^}, repeated 50 times. For ${\mathrm{SPICES}}_{\mathrm{LR‐W}}^{\mathrm{HT}}$ , the same 50 random starting points were used, but no repetitions were performed due to computational costs, resulting in 50 runs. In all cases, the performance is assessed using (i) the median number of unique found conformers required to find a solution and (ii) the 100-S-R, defined as the percentage of trajectories that found a solution in fewer than 100 unique found conformers.

Notably, similar performance is found for both the high- and low-energy targets. The median is 33 for the low-energy target and 41 for the high-energy case, while the 100-S-R value is around 90%. This demonstrates that ${\mathrm{SPICES}}_{\mathrm{LR‐W}}^{\mathrm{HT}}$ is not completely, but largely, independent of energy-based criteria in finding the conformer corresponding to the target spectrum.

As a final benchmark, ${\mathrm{SPICES}}_{\mathrm{LR‐W}}^{\mathrm{HT}}$ is tested against the experimental spectrum of FF. Because of the highlighted low performance of PBE (see Section 4.1), we discuss here only the results obtained with B3LYP. Due to the significantly higher computational cost, we select only 10 starting conformers. The results are shown in the last line of Figure 5. Given the limited data, caution must be exercised. In particular, a direct quantitative comparison with $A‐{\mathrm{SPICES}}_{\mathrm{LR‐W}}^{\mathrm{HT}}$ , which is based on a much larger statistical sample, is not possible. Nonetheless, the impressive median of just 22 and a 100-S-R of 100% allow us to conclude that the implemented approach is effective at efficiently identifying the desired solution.

## 5 General Discussion, Conclusions, and Outlook

The SPectroscopy Informed Configurational Enhanced Sampling (SPICES) method introduced here has been shown to be effective and efficient in identifying the structure corresponding to a specific experimental spectrum.

For our benchmark system, diphenylalanine (FF), the conformation corresponding to the experimental gas-phase spectrum is identified with a median of 22 spectral calculations and a 100-S-R of 100% across test runs, despite the full conformational space containing more than 400 minima. A more general and extensive theory-on-theory benchmark, which also includes high-energy conformers as targets, shows a median of 37 and a 100-S-R above 90%.

Because SPICES requires no prior knowledge, the number of computed spectra is substantially lower than what is typically needed to construct a function Q(I) using, for example, machine-learning approaches (more than 200 spectra). This makes SPICES particularly advantageous for systems where no pretrained data is available. Notably, SPICES does not require *a priori* uniqueness of the solution and can sample multiple valid solutions when distinct structures produce similar spectra, as demonstrated in preliminary tests. In this context, it would be worthwhile to further explore, in future work, the possibility of accurately characterizing the distributions underlying the mixture spectra.

An additional advantage of SPICES is that it is driven by spectroscopic information and is largely independent of the relative stability of the target structure, in contrast with approaches that prioritize sampling based on energy criteria. No refinement with a high-level (expensive) quantum chemistry method is required to obtain the correct ranking of the minima, and no assumption about the energy window in which the solution must fall is required. Indeed, SPICES demonstrates comparable efficiency and, therefore, computational cost for high- and low-energy targets. The level of the theory for the molecular dynamics (MD) module and the spectral calculation module of SPICES can be chosen independently. For the MD, the only requirement is that the chosen level of theory qualitatively captures the positions of the minima on the potential-energy surface. Quantitative agreement with higher-level methods or the correct energetic ranking of the minima is not required. For well-behaved cases, this feature enables the use of low-cost semiempirical methods in MD. For more challenging systems, SPICES’ high efficiency (in the case of FF, the average trajectory is 120 ps) makes the use of GGA functionals feasible for medium-sized systems.

The spectra can instead be computed independently at a higher level of theory. In this respect, accurately predicting spectra is critical to the SPICES performance. Nevertheless, our tests indicate that the optimized scaling factor strategy renders SPICES relatively robust to noise in the experimental spectrum and to minor discrepancies between theory and experiment. In typical applications, the use of hybrid functionals with polarized basis sets in combination with harmonic frequency calculations is sufficient. If greater accuracy is required, anharmonic approaches, such as VPT2, can be employed within SPICES. The reduced number of spectra required by SPICES makes this approach feasible for small-to-medium-sized systems.

Overall, SPICES can be regarded as an effective and computationally efficient strategy for solving the inverse spectral-to-structure problem. The SPICES performance has thus far been critically evaluated only for a single-molecule system. Future studies should expand this assessment to clusters and condensed-phase systems to establish broader applicability; however, no major obstacles are anticipated.

For now, SPICES has been tested only in the context of infrared adsorption spectroscopy. However, the approach is general and can, in principle, be applied to any spectroscopic technique, provided that (i) a computational framework exists to simulate the spectrum and (ii) the spectrum contains sufficient structural information. Exploring its performance across a broader range of spectroscopic methods, therefore, represents an interesting direction for future work.

Although SPICES already demonstrates excellent performance, there remains scope to extend its functionality and further enhance its capabilities. A key component of SPICES is the history-dependent displacement field. At present, the most effective approach appears to be probability-weighted linear regression. In future work, it would be interesting to explore alternative regression models, such as random forests or small neural networks, to improve the overall predictive performance. In particular, it will be interesting to test whether these other prediction models could help with some of the edge-case trajectories where the system finds itself trapped in the wrong part of the PES. Within the same scope, it would be valuable to investigate integrating SPICES with established enhanced-sampling molecular dynamics techniques, such as metadynamics and replica-exchange methods. Because SPICES is formulated within the framework of biased molecular dynamics, it can be readily combined with other approaches in a synergistic manner.

## Supplementary Material



## Data Availability

The manuscript introduces a new theoretical method. The main text and the Supporting Information describe the underlying theory, algorithmic procedure, and implementation strategy in detail. The key derivations are also provided. The data sets containing the FF molecular structures and spectra used to test the method are available at 10.5281/zenodo.21237345.

## Notes

Additional details on the use of periodic boundary conditions, symmetry, and the jumping of minima for A-SPICES; additional strategies and modules for SPICES, supported by benchmarking (PDF)
