# Supplementary material for: SPectroscopy Informed Configurational Enhanced Sampling (SPICES)
Source: J Chem Theory Comput. 2026 Oct 23;22(21):11471–83. doi: 10.1021/acs.jctc.6c01326 (PMC13757752; doi:10.1021/acs.jctc.6c01326)
Supplement: Supplementary file 1 [file ct6c01326_si_001.pdf]

Supporting Information: SPectroscopy Informed Configurational Enhanced Sampling (SPICES)

Roel van de Ven, Herma Cuppen, and Daria Ruth Galimberti*

Institute for Molecules and Materials, Radboud University, Heyendaalseweg 135, 6525 AJ Nijmegen, The Netherlands

E-mail: daria.galimberti@ru.nl

S0. List of abbreviations

$\boldsymbol{s_n}$: history-dependent configurational displacement field or shortened to history-dependent displacement field.

R: Initialization using a set of random biases for this first move, and the standard $\boldsymbol{s_n}$ protocol after.

HT: High temperature sampling initialization.

LR: History-dependent displacement field based on linear regression.

LR-W: History-dependent displacement field based on linear regression + geometrical criteria weights.

LR-LCCS: History-dependent displacement field based on linear regression + LCCS criteria weights.

MC: Monte Carlo strategy.

SPICES: Normal SPICES.

A-SPICES: SPICES in which the MD has been substituted by artificial propagation using a database of pre-computed spectra.

100-S-R: The percentage of runs that find a solution within 100 unique explored conformers, i.e., high-level DFT calculations.

All the tests with A-SPICES have been run both with the set of structures optimized at the PBE-D3(BJ)/6-31G(d,p) and at the B3LYP-D3(BJ)/6-311G(d,p) level of theory (see Section 3 of the main manuscript for more information on the computational setup). To simplify notation, in the following discussion, PBE-D3(BJ)/6-31G(d,p) will be abbreviated to **PBE**, and B3LYP-D3(BJ)/6-311G(d,p) to **B3LYP**.

S1. Periodic boundary conditions and symmetry

As discussed in the main manuscript, as a test system, we selected gas-phase diphenylalanine (FF). For SPICES, we select nine dihedral angles (see main text Figure 2) as the set of coordinates $\{q_i\}$, whose values $\{Q_i\}$ uniquely identify the system structure. Cyclic periodic boundary conditions must be taken into account in building the history-dependent displacement field, $\boldsymbol{s_n}$, and in recognizing the minima of the Potential Energy Surface (PES). For this purpose, both the structural comparison and the regression used to construct $\boldsymbol{s_n}$ are performed not on the dihedral angle values θ_i directly, but on their transformed representations $\sin(n\theta_i)$ and $\cos(n\theta_i)$, where n denotes the n-fold symmetry associated with θ_i. For example, in the case of benzene rings, $n = 2$, i.e., two-fold symmetry. Additionally, to prevent the inclusion of permutation-equivalent structures in the reference set, we impose that the tracked torsional angle remains within a specific range with periodicity $\frac{2\pi}{n}$, e.g., $[0, \pi]$ in the case of twofold symmetry. If this condition is violated, the associated atom indices are swapped to restore it.

Finally, the amine group represents a special case. Likely due to an underestimation of the PES barrier along the H-N-H angle coordinate at the DFTB level, some trajectories exhibit occasional artificial, rapid roaming of one amide hydrogen atom around the nitrogen atom, leading to an inversion of the two hydrogen atoms. When such behavior is detected, the hydrogen atoms are swapped to prevent including structures that differ only by permutation within the dataset.

S2. Identification of Minima Regions in the MD Simulation

As described in Section 2.2 of the main manuscript, the MD simulation used to steer the system through conformational space proceeds in a series of macrosteps (Γ_i). At the end of each macrostep, candidate minima are identified according to the following protocol. The potential energy at each point along the macrostep is compared with those of the conformations sampled within the preceding and subsequent 50 fs. A point is identified as a candidate minimum or maximum if its potential energy is, respectively, the lowest or highest within this 100 fs window. The resulting list of candidate minima and maxima is then filtered to ensure that it starts with a maximum and alternates between maxima and minima. When neighboring minima or maxima are identified, only the most extreme point is retained, and the other is discarded. For each remaining minimum, its energy is compared with that of the preceding maximum. If the energy decreases by at least 0.015 Ha, the minimum is classified as a relevant temporal minimum, and the corresponding structure is passed to the conformer recognition procedure described in Section 2.3 of the main text.

S3. Frequencies scaling factor

As discussed in the main manuscript, because the optimal scaling factor for a system is not always known *a priori*, a strategy that optimizes it during SPICES for the specific system under study has been designed. For each $\boldsymbol{I}^{\text{ref}}$, the scaling factor is optimized via a grid search to maximize the spectral similarity score with the target spectrum. In this way, each structure is given the best opportunity to match the experimental spectrum. This strategy will be referred to as scale=opt in Figure S1.

As a sanity check of this protocol, we compared the results obtained with the adaptive approach to those from a fixed scaling–factor strategy. In the latter case, we tested both the standard tabulated scaling factors from the CCCBDB database[1] (0.986 and 0.966 for PBE and B3LYP, respectively) and scaling factors specifically optimized for the benchmark system (1.001 and 0.975 for PBE and B3LYP, respectively).

The performance of different scaling-factor strategies was critically assessed using 50 random initial points selected from $\{\boldsymbol{Q}^{\text{full}}\}$. The A-$\text{SP}^{\text{HT}}_{\text{LR-W}}$ procedure was then run 50 times for each starting point, resulting in a total of 2500 simulations.

As can be seen from Figure S1, in the case of the B3LYP set, similar performance is observed for the scale=opt strategy and the optimal fixed scaling factor, both yielding a median value of approximately 33 unique found conformers and a 100-S-R close to 99%. Notably, the slight discrepancy between the optimal scaling factor (0.975) and the CCCBDB value (0.966) translates into measurable performance differences. With the fixed CCCBDB scaling factor, the median increases to 38, and the 100-S-R decreases to 95%. These latter results, although still satisfactory, suggest that the fixed scaling–factor strategy should be preferred only when the scaling factor is known with high confidence.

These results are confirmed by the PBE test case. Some caution is required when analyzing the data. For the B3LYP level of theory, which provides high-quality data for this molecule[2], the MP2 global minimum is the conformer with the best LCCS score. Moreover, the distance in score between the best (0.902) and the second (0.893) is large enough to

confidently distinguish between the two. In the case of PBE, for which the spectra are qualitatively accurate but not quantitatively, this is not the case. Specifically, the MP2 global minimum conformation ranks 24th with an LCCS score of 0.812, while the best-ranked conformer has a score of 0.856. Therefore, for this specific test, the values of $\{Q_i\}$ are also monitored to ensure that A-SPICES terminates only upon identifying the correct conformer. Analyzing the effect of different scaling factor strategies, we observe similar results to B3LYP. The poorest performance is obtained using the tabulated value (0.986), yielding a median of 107 unique found conformers and a 100-S-R of 46.2%. Slightly better results are achieved with the scaling factor explicitly optimized for the system (1.001), giving a median of 101 and a 100-S-R of 49%. Interestingly, the scale=opt strategy provides a substantial improvement, reducing the median to 78 and increasing the 100-S-R to 66%.

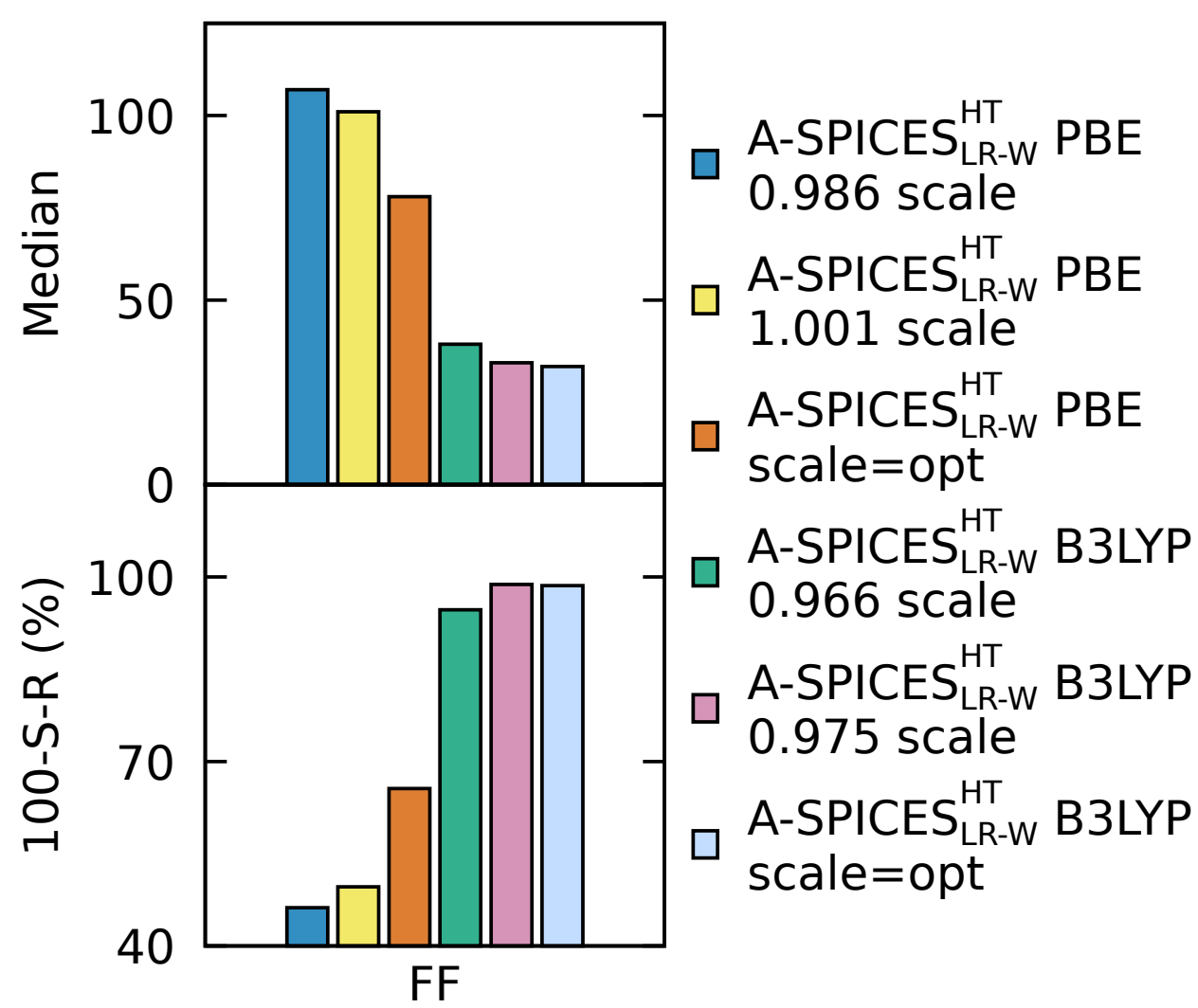

Figure S1: Performance of A-SPICES$^{\mathrm{HT}}_{\mathrm{LR\text{-}W}}$ in assigning the experimental spectrum of FF, using either PBE-D3(BJ)/6-31G(d,p) or B3LYP-D3(BJ)/6-311G(d,p) for spectral calculations (PBE and B3LYP in the figure), and employing different scaling factor strategies: the tabulated scaling factors from CCCBDB database (0.986 and 0.966 for PBE and B3LYP, respectively), the optimized scaling factors for the system (1.001 and 0.975 for PBE and B3LYP respectively), and the scale=opt strategy. In total, 2500 runs were performed using 50 random starting points from $\{\boldsymbol{Q}^{\mathrm{full}}\}$, each repeated 50 times.

S4. A-SPICES jumping between minima.

Within A-SPICES the MD step of SPICES is substituted by a walker that jumps directly between PES minima using the rules outlined below.

At each nth iteration, the walker's move is determined by the history-dependent displacement field, $\boldsymbol{s_n}$, which is defined using the $n-1$ previously explored minima, as in the SPICES procedure. In this case, however, $\delta\boldsymbol{\Omega}^n$ is fixed to $\frac{1}{20}\boldsymbol{s_n}$. For a maximum of 20 steps, the structure is propagated according to $\boldsymbol{Q}^n(t+1) = \boldsymbol{Q}^n(t) + \delta\boldsymbol{\Omega}^n$. After each step, A-SPICES verifies whether a new conformation has been "identified", i.e., whether the move to the next minimum occurs. This requires the current structure $\boldsymbol{Q}^n(t+1)$ to be sufficiently different from the reference set $\{\boldsymbol{Q}^{\text{ref}}\}$ of already explored conformations, i.e., at least one torsional angle must differ by more than 1.2 rad ($\sim 69°$). Simultaneously, $\boldsymbol{Q}^n(t+1)$ must be sufficiently close to a structure in $\{\boldsymbol{Q}^{\text{full}}\}$ that is not included in $\{\boldsymbol{Q}^{\text{ref}}\}$, which is enforced by requiring the maximum torsional-angle deviation to be smaller than 2.4 rad ($\sim 138°$). If multiple structures satisfy these criteria, the n-th new conformation is selected as the one closest to the trial structure $\boldsymbol{Q}^n(t+1)$, i.e., the one with the smallest maximum torsion-angle deviation. If after 20 steps a new minimum has still not been identified, the closest conformer to $\boldsymbol{Q}^n(t=20)$ in $\{\boldsymbol{Q}^{\text{full}}\}$ but not in $\{\boldsymbol{Q}^{\text{ref}}\}$ is selected as the new minimum.

S5. history-dependent configurational displacement field and its initialization

All the tests discussed in the following have been obtained using A-SPICES, with a target computed at the same level of theory (either PBE or B3LYP) used for SPICES in the specific test. For all the cases, the performance is evaluated over 12500 runs, comprising 5 random targets from $\{\boldsymbol{I}^{\text{full}}\}$ and 50 random starting points from $\{\boldsymbol{Q}^{\text{full}}\}$, with each start–target pair repeated 50 times.

S5.1 Effect of the theory on the performance

As an initial validation step, Figure S2 presents a critical assessment of the internal performance of A-SPICES across various configurations by comparing results obtained with the PBE and B3LYP levels of theory, using computed targets derived from the same dataset used for testing. Despite minor differences in the results, consistent trends are observed, confirming the reliability of the implemented strategies.

Consider now the performance of SPICES when the target is the experimental spectrum (Figure S1). As discussed in the main manuscript, the choice of level of theory is shown to be critically important for the performance of SPICES when targeting experimental spectra. For example, the best performance of PBE yields a median of only 78 unique found conformers and a 100-S-R of 66%, while B3LYP finds the solution with a median of 32 and a 100-S-R of 99%.

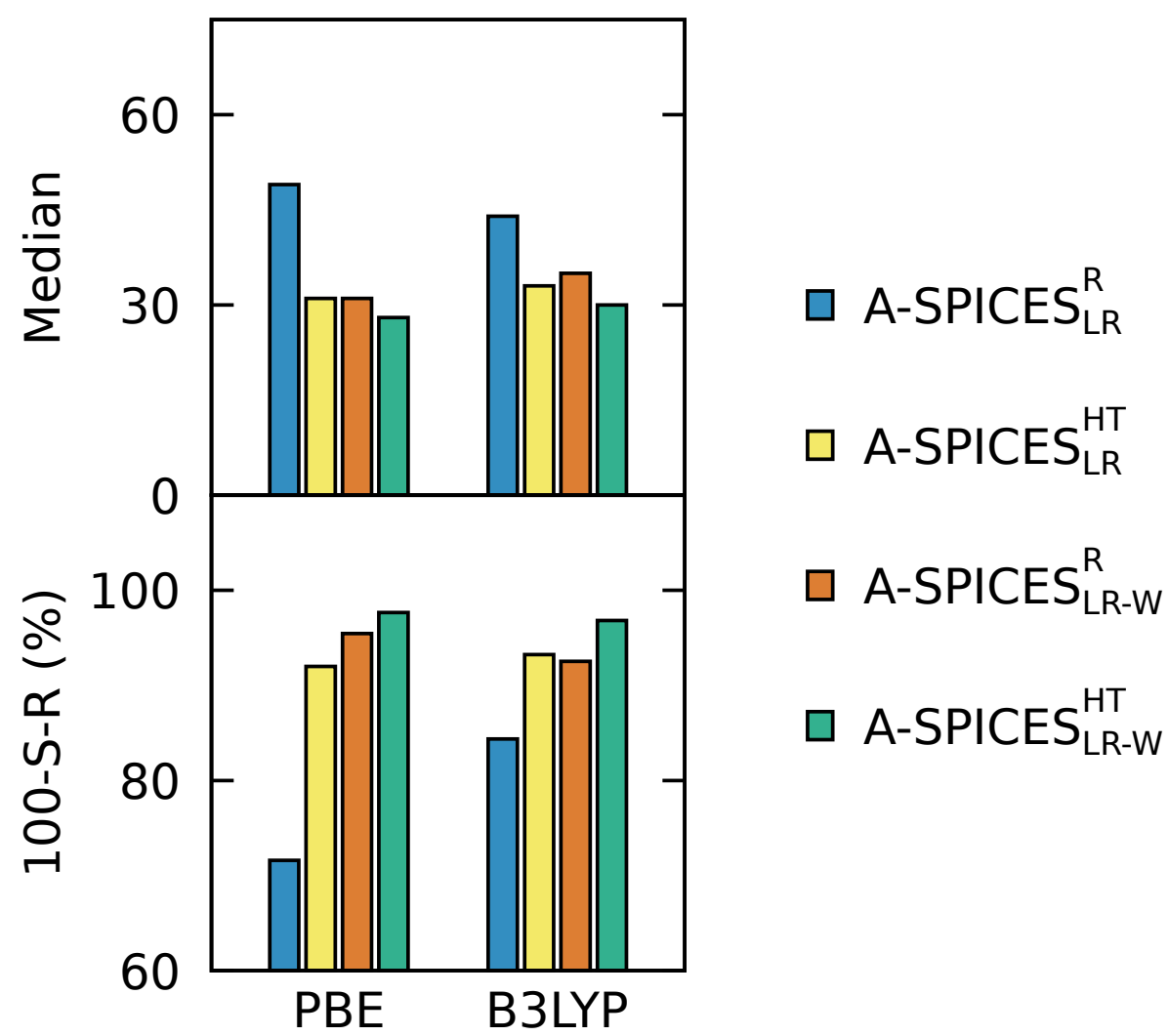

Figure S2: Performance of A-SPICES in different configurations (A-SPICES$^{\mathrm{R}}_{\mathrm{LR}}$, A-SPICES$^{\mathrm{HT}}_{\mathrm{LR}}$, A-SPICES$^{\mathrm{R}}_{\mathrm{LR\text{-}W}}$, and A-SPICES$^{\mathrm{HT}}_{\mathrm{LR\text{-}W}}$) using either PBE or B3LYP. Performance is evaluated over 12500 runs, comprising 5 random targets from $\{\boldsymbol{I}^{\mathrm{full}}\}$ and 50 random starting points from $\{\boldsymbol{Q}^{\mathrm{full}}\}$, with each start–target pair repeated 50 times.

S5.2 Selection of the starting conformer with HT

As discussed in section 2.5 of the manuscript, different strategies can be used in the HT module to select the conformer after an initial high-temperature sampling. In Figure S3, the effect of the choice is critically assessed. The default approach—based on selecting the structure with the highest LCCS score relative to the target spectrum (A-SPICES$^{\text{HT}}_{\text{LR}}$)—is compared against two alternative strategies: using the original starting structure (A-SPICES$^{\text{HT-S}}_{\text{LR}}$) and using the final structure obtained after high-temperature sampling (A-SPICES$^{\text{HT-E}}_{\text{LR}}$). The different approaches yield nearly identical results, suggesting that the improved performance observed with high-temperature sampling primarily arises from enhanced exploration of the conformational space rather than from differences in the initial starting point.

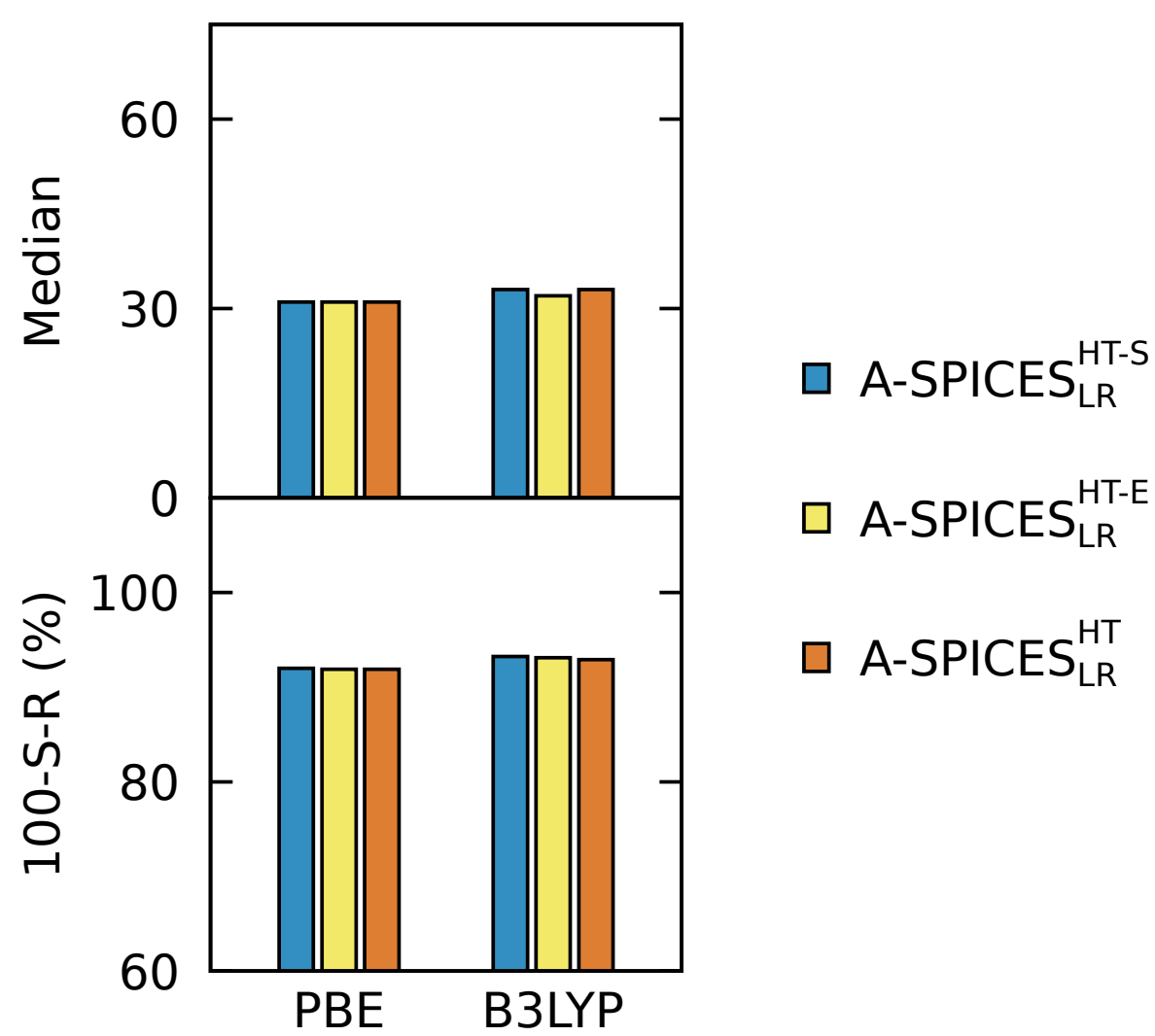

Figure S3: Performance of A-SPICES$^{\text{HT}}_{\text{LR}}$ using different strategies for selecting the conformer after the high-temperature sampling using either PBE or B3LYP. Performance is evaluated over 12500 runs, comprising 5 random targets from $\{\boldsymbol{I}^{\text{full}}\}$ and 50 random starting points from $\{\boldsymbol{Q}^{\text{full}}\}$, with each start–target pair repeated 50 times.

S5.3 Monte Carlo approach

By default, SPICES accepts every move, i.e., the newly generated structure is always used as the starting point for the subsequent iteration. A Monte Carlo/Metropolis approach, in which moves are not always accepted, has also been tested. The acceptance criterion for each move has been based on the LCCS score. Two strategies have been evaluated for this. In the first strategy (MC1) a move is always accepted if the LCCS score improves relative to the previous step. If the score decreases, the move is accepted with a probability of 50%. In the second strategy (MCn) the best LCCS score is used for the comparison. A move is always accepted if the LCCS score improves relative to the best score. If the score decreases, the move is accepted with a probability of 50%. If it is not accepted, SPICES moves back to the structure with the highest LCCS score. As can be seen in Figure S4, on average, neither MC1 nor MCn approaches result in a lower median or an increased success rate.

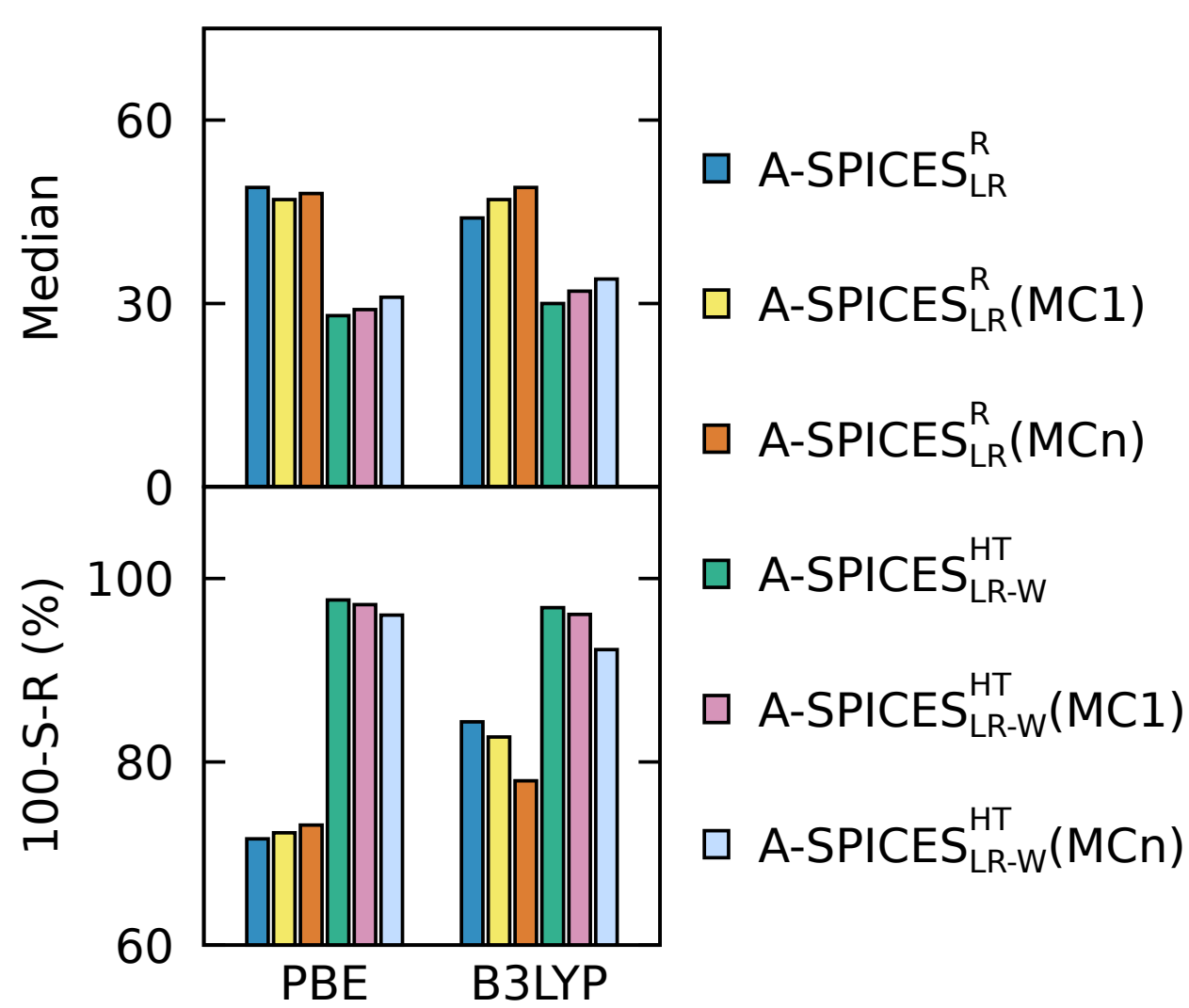

Figure S4: Performance of A-SPICES using the default strategy (every move is accepted), or making use of the Monte Carlo/Metropolis approach in the MC1 and MCn variants (see text above for details) using either PBE or B3LYP. Performance is evaluated over 12500 runs, comprising 5 random targets from $\{\boldsymbol{I}^{\text{full}}\}$ and 50 random starting points from $\{\boldsymbol{Q}^{\text{full}}\}$, with each start–target pair repeated 50 times.

S5.4 LCCS probability-based weights

As discussed in the main manuscript, the space we are dealing with is inherently nonlinear, raising concerns about whether assuming a linear form is overly crude or simplistic for defining $\boldsymbol{s_n}$. Starting from the observation that the closer two points, α and β, are in a space, the more valid the assumption of local linearity becomes, i.e., the higher is the probability p_α^β that the information from the α reference is still relevant for understanding the correct move of a walker starting from $\boldsymbol{Q}^\beta$ adopting a linear approximation. In the default strategy (SPICES$_{\text{LR-W}}$), the probability is based on the distance between the two structures in the $\boldsymbol{Q}$ space. We also tested the possibility of using the distance in the spectral space, measured by the LCCS score (SPICES$_{\text{LR-LCCS}}$). The results of this test are shown in Figure S5. Similar results are obtained by A-SPICES$^{\text{R}}_{\text{LR-LCCS}}$ and A-SPICES$^{\text{R}}_{\text{LR-W}}$, with perhaps a slight advantage for the LCCS-based weights, as the medians are lowered by one for both PBE (30 vs 31) and B3LYP (34 vs 35). Similarly, the 100-S-R improves for both, with increases from 96% to 97% for PBE and from 93% to 95% for B3LYP. If the initial high temperature sampling strategy is also used, the two configurations (A-SP$^{\text{HT}}_{\text{LR-LCCS}}$ vs A-SP$^{\text{HT}}_{\text{LR-W}}$) show no significant differences. Because LCCS-based probability weights do not provide a substantial advantage — at least for the case tested — and are more difficult to set up (in particular, selecting an appropriate value of σ is less intuitive), the geometry-based criterion was chosen as the default.

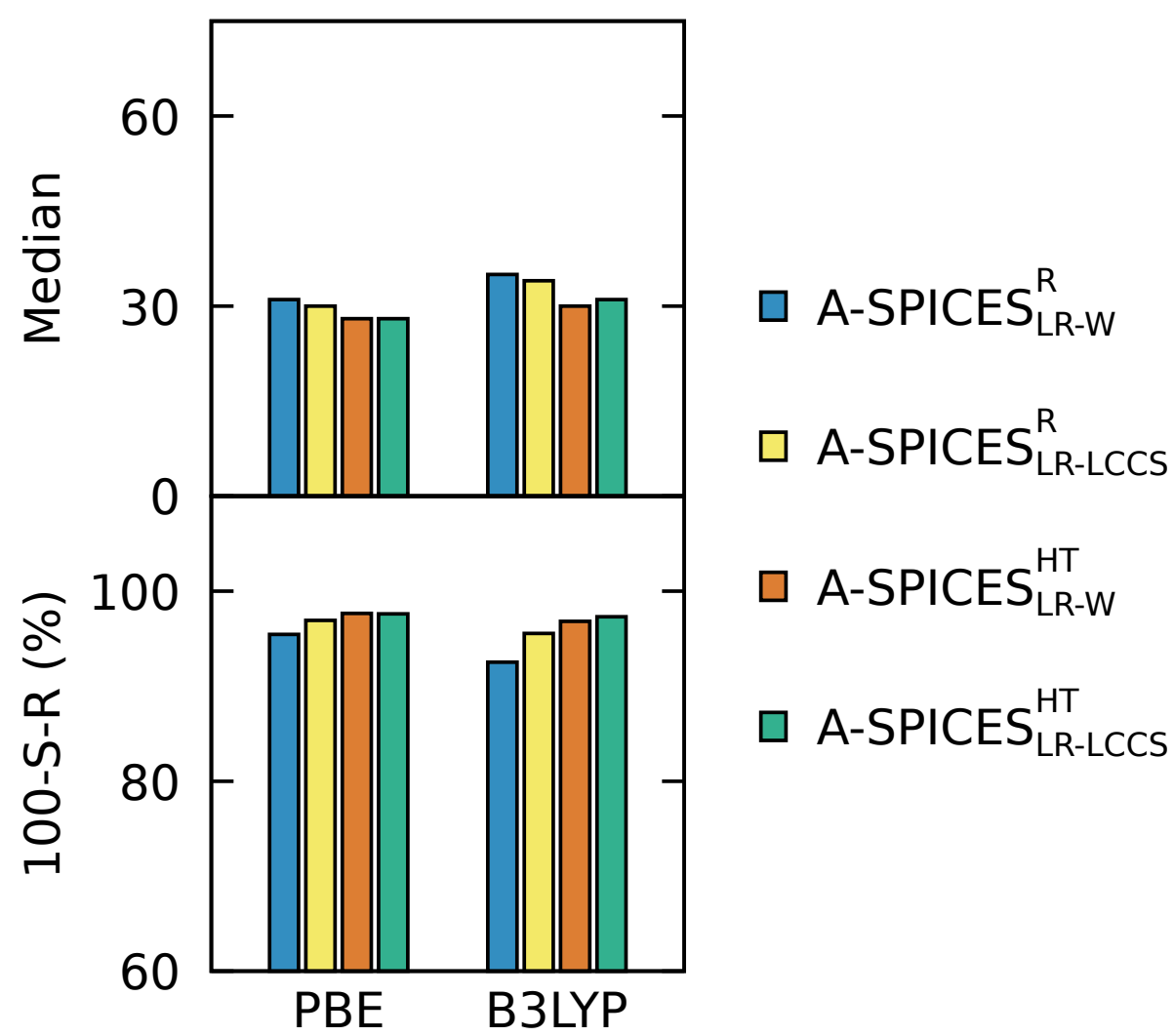

Figure S5: Performance of A-SPICES using different strategies for the weights: either geometrical distances (W) or spectral distances (LCCS) using the LCCS score ($\sigma^2 = 0.1$) with either PBE or B3LYP. Performance is evaluated over 12500 runs, comprising 5 random targets from $\{\boldsymbol{I}^{\text{full}}\}$ and 50 random starting points from $\{\boldsymbol{Q}^{\text{full}}\}$, with each start–target pair repeated 50 times.

S5.5 Alternatives to incorporate weights

As discussed in section 2.4 of the main manuscript, the default strategy ($\mathrm{SPICES}_{\text{LR-W}}$) for incorporating the weights w_β^α into the regression model is to introduce $\boldsymbol{X}_n$ the matrix that collect the $\{w_i^n \tilde{\boldsymbol{I}}^i\}$ elements and $\boldsymbol{Y}_n$ the matrix that collects the corresponding $\{w_i^n \boldsymbol{Q}^i\}$, and build $\boldsymbol{s_n}$ as:

$$\boldsymbol{s_n}(\boldsymbol{I}^n, \boldsymbol{I}^*, \boldsymbol{Q}^n) = \boldsymbol{B}_n^T \tilde{\boldsymbol{I}}^* - \boldsymbol{Q}^n \tag{1}$$

with

$$\boldsymbol{B_n} = (\boldsymbol{X}_n^T \boldsymbol{X}_n)^{-1} \boldsymbol{X}_n^T \boldsymbol{Y}_n \tag{2}$$

In Figure S6, we compare the performance of $\mathrm{SPICES}_{\text{LR-W}}$ with that obtained by incorporating the weight into the loss function, namely the Root Mean Square Error (RMSE) as in a standard weighted linear regression:

$$\mathrm{RMSE} = \sqrt{\frac{1}{m} \sum_{i=1}^{m} w_i^n (\hat{y} - y_i)^2}, \tag{3}$$

Both in the case of the structure-based and LCCS-based weights, the performance of this latter strategy ($\mathrm{SPICES}_{\text{LR-W-X}}$ and $\mathrm{SPICES}_{\text{LR-LCCS-X}}$, respectively) is substantially worse than our default strategy.

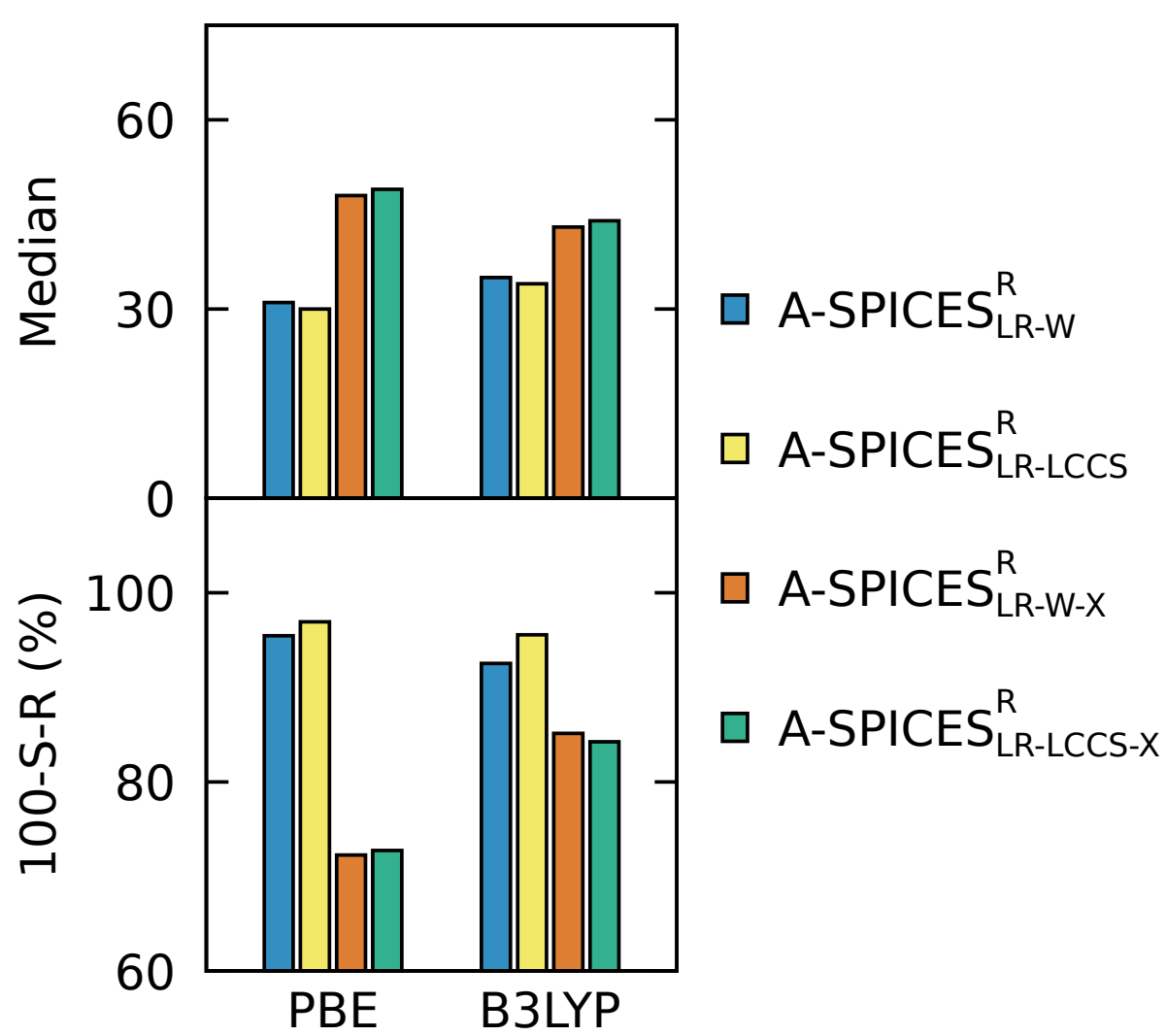

Figure S6: Performance of A-SPICES using different strategies of applying weights: directly (SPICES$^{\text{R}}_{\text{LR-W}}$ and SPICES$^{\text{R}}_{\text{LR-LCCS}}$) or on the loss function (SPICES$^{\text{R}}_{\text{LR-W-X}}$ and SPICES$^{\text{R}}_{\text{LR-LCCS-X}}$) for both the geometry-based and the LCCS-based weights using either PBE or B3LYP. Performance is evaluated over 12500 runs, comprising 5 random targets from $\{\boldsymbol{I}^{\text{full}}\}$ and 50 random starting points from $\{\boldsymbol{Q}^{\text{full}}\}$, with each start–target pair repeated 50 times.

S6. Multiple solutions test run

To evaluate the ability of SPICES to sample multiple solutions, an additional test was conducted using A-SPICES at the B3LYP level with the experimental spectrum as the target. The acceptance threshold for matching the experimental spectrum was reduced from 0.902 (B3LYP(1)) to 0.893 (B3LYP(3)), yielding three spectra in the set classified as matches. For these tests, 50 random starting points from $\{\boldsymbol{Q}^{\text{full}}\}$ have been selected, and A-SPICES$^{\text{HT}}_{\text{LR-W}}$ has been run 50 times for each, resulting in a total of 2500 simulations. The results of these tests are shown in Figure S7. All three structures are identified by SPICES. The structure with the highest LCCS score compared to the experimental spectrum is the most frequently sampled (40.1% of cases). The other two instead are found 27.7% and 32.2% of the time.

	Median	100-Success-rate (%)	#1 LCCS (%)	#2 LCCS (%)	#3 LCCS (%)
A-SPICES$^{\text{HT}}_{\text{LR-P}}$ B3LYP(1)	32	98.6	100.0	0.0	0.0
A-SPICES$^{\text{HT}}_{\text{LR-P}}$ B3LYP(3)	22	99.8	40.1	27.7	32.2

Figure S7: Performance of A-SPICES$^{\text{HT}}_{\text{LR-W}}$ in assigning the experimental spectrum of FF, using B3LYP-D3(BJ)/6-311G(d,p) for spectral calculations (B3LYP in the figure) and accepting as solution only the best match, B3LYP(1), or the three best matches, B3LYP(3). Performance is evaluated over 2500 runs, 50 random starting points from $\{\boldsymbol{Q}^{\text{full}}\}$, repeated 50 times.

References

(1) Irikura, K. K.; Johnson, R. D.; Kacker, R. N. Uncertainties in Scaling Factors for ab Initio Vibrational Frequencies. *The Journal of Physical Chemistry A* **2005**, *109*, 8430–8437.

(2) van de Ven, R.; Cuppen, H. M.; Galimberti, D. R. Influence of the computational methods in the conformational assignment of experimental infrared spectra. *Vibrational Spectroscopy* **2025**, *141*, 103863.
